# Data-driven and feature-sifting based state-of-health estimation for li-ion batteries using KPCA and NRBO-optimized transformer-LSTM hybrid model

**DOI:** 10.1371/journal.pone.0359015

**Published:** 2026-10-05

**Authors:** Xiangcai Luo, Jinwei Chen, Haijiang Zhang, Chunhua Hu, Jinyong Zhao, Fei Xu, Rui Zhen, Chengchen Deng, Xi Chen

**Affiliations:** 1 State Grid Neijiang Power Supply Co., Ltd., Neijiang, Sichuan, China; 2 College of Electrical Engineering and New Energy, China Three Gorges University, Yichang, Hubei, China; Sunway University, MALAYSIA

## Abstract

Electrochemical energy storage technologies, such as lithium-ion batteries, are widely used in various scenarios, but their capacity, lifespan, and state-of-health (SOH) are susceptible to numerous influencing factors. To improve the accuracy of state-of-health (SOH) prediction for lithium-ion battery cells, this paper builds up a feature-based analysis framework, and proposes an enhanced predicting model, which integrates the principles of incremental capacity analysis (ICA) and differential voltage analysis (DVA), and the ideas of kernel principal component analysis (KPCA), and Pearson correlation analysis. The former ones are used to extract a set of health related features (HFs) from the filtered incremental capacity (IC) and differential voltage (DV) curves of batteries, while the latter ones are employed to eliminate redundant features. Furthermore, this work bridges the gap between the charging data of the cells and their SOH by building up a Newton-Raphson-based optimization Transformer-LSTM (NRBO-Transformer-LSTM) model, where NRBO algorithm is introduced to find out the optimized number of attention heads, learning rates and the regularization coefficient. Experimental validation on the NASA and CALCE battery datasets shows that, with 70% of the data used as the training set, the proposed method achieves maximum mean absolute errors (MAE) of 0.0084 and 0.0071, and root mean square errors (RMSE) of 0.0091 and 0.0089, respectively, significantly outperforming multiple benchmark algorithms in both prediction accuracy and stability.

## 1. Introduction

Electrochemical energy storage components, represented by lithium-ion batteries, are highly favored in renewable energy power systems due to their specific capacity characteristic, their ability to smooth energy fluctuations, and their excellent cycling performance [1]. However, due to various health-related factors such as the variations of electrolyte concentration, changing working temperatures, and charge and discharge rates, the SOH of lithium-ion battery cells can be altered, which reduce the reliability and economic viability of projects in large-scale applications such as energy storage in the power grids [2–5]. Therefore, accurately predicting the SOH of lithium-ion battery cells is essential for the design of the thermal management, charge-discharge strategies, ensuring component performance, extending cell lifespan, and ensuring safety [6].

As the SOH of battery cells cannot be measured directly, numerous studies have focused on its estimation and prediction, with common approaches including direct measurement methods, model-based techniques, and data-driven strategies, each with their own pros and pons, and applicable scenarios.

Generally, direct measurement methods measure characteristic parameters that reflect battery aging at first, such as capacity, internal resistance, and impedance, and then analyze and calculate the corresponding SOH. The advantage of this type of method is that it can intuitively correlate the measurement results with the battery SOH. However, the requirements for measurement results have also brought negative impacts, especially on the requirements and limitations of measurement environments, resulting in measurement results that cannot fully reflect all influencing factors [7]. For example, literature [8] applies a short-duration DC pulse to measure internal resistance, successfully distinguishing batteries at different SOH levels. In contrast, literature [9] uses electrochemical impedance spectroscopy (EIS), but finds that temperature and state of charge (SOC) variations introduce measurement errors, compromising the accuracy of SOH estimation.

To address the shortcomings of direct measurement methods and reveal the factors affecting battery SOH at the mechanism level in an all-round way, several model-based analysis techniques have emerged. Typical SOH prediction models include equivalent circuit models (ECMs) and electrochemical models. Currently, the ECMS used for estimating the SOH of lithium-ion batteries primarily consist of standard and improved structures. Traditional models, such as the first-order Thevenin model and the second-order RC model, represent battery ageing through changes in parameters such as ohmic resistance or multiple polarisation parameters, and are applicable under general operating conditions [10,11]. Based on this foundation, recent studies have further expanded the ECM architecture and application scenarios. Reference [12] presents a new ECM based on an RL network, where the traditional RC branch is replaced by two parallel resistor-inductor (R-L) branches to examine the current decay behaviour in the constant-voltage (CV) charging stage. By decoupling the fast and slow dynamic components, this method obtains a time constant that has high correlation with capacity degradation, thereby improving the sensitivity and accuracy of SOH estimation during CV charging to a great extent. Reference [13] presents an improved ECM (E-ECM) that employs a standard second-order RC structure, supplemented by a first-order inertial block, to detect the concentration gradient between the electrode surface and the bulk caused by solid-phase diffusion. The aforementioned model can achieve higher voltage prediction accuracy when the SOC is low, thus its parameters are closer to representing the non-linear electro-chemical characteristics of the battery degradation. A large number of established electrochemical model-based SOH estimation approaches have progressed from high-fidelity but computationally expensive pseudo-two-dimensional (P2D) models to simplified frameworks balancing mechanistic fidelity and practicability. Some studies have used a fractional-order transfer function to approximate solid-phase diffusion and constructed a single-electrode model that only includes the positive electrode, using parameters to represent capacity decay and internal resistance increase, respectively [14]. Literature [15] incorporates SEI growth and stress-induced particle cracking mechanisms into a single-particle model (SPM) to reveal the synergistic ageing effects of active lithium loss and increased interfacial impedance. In addition, fully coupled multi-physics P2D ageing models can comprehensively integrate electrochemical, thermal and mechanical processes to reproduce capacity and power loss caused by simultaneous SEI film growth, lithium electrodeposition and active material loss at different temperatures [16]. Among them, the most recent advancement is the FOMeA model that integrates fractional-order Padé approximation and a two-state electrolyte system to significantly improve computational efficiency and explicitly incorporate SEI growth and lithium plating/stripping mechanisms, including the formation of “dead lithium.” This method can dynamically update the resistance of SEI film and lithium-plating, thus accurately reflecting the battery’s aging characteristics at all stages of its entire life cycle, such as capacity attenuation, impedance increase, and poor high-rate performance [17]. In summary, the direction of development for model lightweighting by rationally simplifying the structure has been validated; meanwhile, it still retains key features such as electrochemical reactions to achieve online estimation of battery SOH. A certain degree of the model-based analysis and prediction methods has a certain level universal but there is a direct relationship between the accuracy of prediction of this model complexity. At present, most existing models are developing in the direction of incorporating more elements to better represent various influencing factors. However, the increase in model complexity has made it more difficult to identify model parameters [18].

Given the rapid development of artificial intelligence technology, as one of its typical applications, data-driven predictive methods have gradually become an essential tool for assessing lithium-ion Batteries. Usually, these approaches do not consider the underlying reasons why electrochemical reactions occur in batteries; therefore, batteries are regarded as “black boxes,” and changes in charge and discharge currents are used to estimate their conditions. Using data obtained from tests or public databases as a sample set for training the prediction model; after training, this model can be used for battery SOH predictions. Compared with the two methods mentioned above, a data-driven approach is more appropriate for online or real-time estimation; it has a low dependence on precise special equipment; It can achieve high computational efficiency. Data-driven methods provide another path for estimation. In recent years, many research efforts have been made, and a large number of algorithms have been developed [19–29]. Reference [19] proposes the ResBiGRU-AttnAE model, which has a light structure and high accuracy. It only needs charging-phase features to achieve fast online SOH estimation, making it suitable for embedded deployment; however, it lacks physical interpretability. Reference [20] uses a multi-scale CNN-BiLSTM fusion structure to enhance robustness and noise resistance through the combination of raw sensor signals and time-domain health information, and has shown stable performance in multiple datasets; however, its high computational cost limits real-time implementation on resource-constrained battery management systems (BMS), and some of its features depend on full charge-discharge cycles. Reference [21] proposes a hybrid framework combining a simplified electrochemical model with LSTM-based correction, which provides some physical interpretability and reduces the need for a large number of training data sets; However, this method requires battery chemistry-specific parameter calibration, has weak cross-chemistry generalisation ability, and its system is relatively complex, making it difficult to be applied in practical engineering. Reference [22] proposes an improved TWSVM-based method for joint SOH and RUL estimation, achieving high prediction accuracy across multiple datasets and requiring only the easily obtainable constant-current charging time as input, demonstrating strong engineering practicality; however, it lacks physical interpretability, and its overall computational complexity is relatively high, making it unsuitable for embedded deployment. In general, although the four approaches mentioned above differ in focus-accuracy, efficiency or interpretability-they all have limitations; data quality is required, generalisation performance is lacking in a real environment, and prediction reliability cannot be guaranteed.

To address the aforementioned issues with existing works, this paper presents a data-driven feature-sifting analysis framework for SOH prediction of battery cells. Through analysis of the IC and DV curves to determine the aging behaviour, a new set of HFs is generated. Using Pearson correlation analysis to determine the extent of association between HFs and battery capacity. In addition, to reduce computational complexity, KPCA is applied to the selected health features for dimensionality reduction, and indirect health features are obtained. To enhance prediction results, a Transformer-LSTM Predictive Model with temporal attention mechanisms can be introduced. Some important hyperparameters, such as the number of attention heads, learning rate, and regularisation coefficient, are optimised using a Newton-Raphson-based algorithm at the same time. In this Model, transformer can capture global temporal dependence, LSTM is good at capturing local dynamic characteristics, and attention mechanism pays more attention to the effect of health status. By combining the forward and backward sequence information, the proposed method can achieve high-accuracy prediction of battery SOH, thereby obtaining high-accuracy and strong robustness SOH predictions.

The following are the organisation of this paper: Section II introduces the extraction and optimisation of health features; Section III describes the overall process for estimating SOH; Section IV presents experimental verification results; and Section V draws conclusions.

## 2. Health feature sifting and optimization

### 2.1. Battery datasets

The data used in this paper are sourced from the NASA and CALCE lithium-ion battery cycling aging datasets. Tables 1 and 2 summarize the main characteristics of the selected batteries, including their specifications and operating conditions. Fig 1 show the capacity degradation curves of the selected batteries from the two datasets, respectively. In the NASA dataset, the battery charging process consists of two stages: an initial constant-current (CC) stage followed by a constant-voltage (CV) stage. Specifically, the battery is first charged at a constant current of 1.5 A until the voltage reaches 4.2 V. Subsequently, it enters the constant-voltage charging mode until the charging current drops to 20 mA. During the CC stage, the battery temperature continuously rises to a peak value and then gradually decreases during the CV stage. The CALCE battery aging dataset was collected under standard room-temperature conditions (25 ± 2°C) using a strictly controlled charge-discharge protocol: the charging phase begins with a constant current of 0.5A until the voltage reaches 4.2V, followed by constant-voltage charging until the current drops below 0.05A; the discharge phase employs a constant current of 1A until the voltage decreases to the cutoff voltage of 2.7V.

**Table 1:** The specifications and operational conditions of the NASA batteries under investigation.

Battery Number	Discharge Current	Cut-off Voltage	Ambient Temperature	Rated Capacity	EOL
	/A	/V	/°C	/Ah	/Ah
B0005	2	2.7	24	2	1.40
B0007	2	2.2	24	2	1.45
B0029	4	2.0	43	–	1.65
B0031	4	2.5	43	–	1.70

**Table 2:** The specifications and operational conditions of the CALCE batteries under investigation.

Battery Number	Discharge Current	Discharge cut-off	Charge cut-off voltage	Rated Capacity	EOL
	/A	voltage /V	/V	/Ah	/Ah
CS2_35	1	2.7	4.2	1.1	0.77
CS2_36	1	2.7	4.2	1.1	0.77

**Fig 1:**
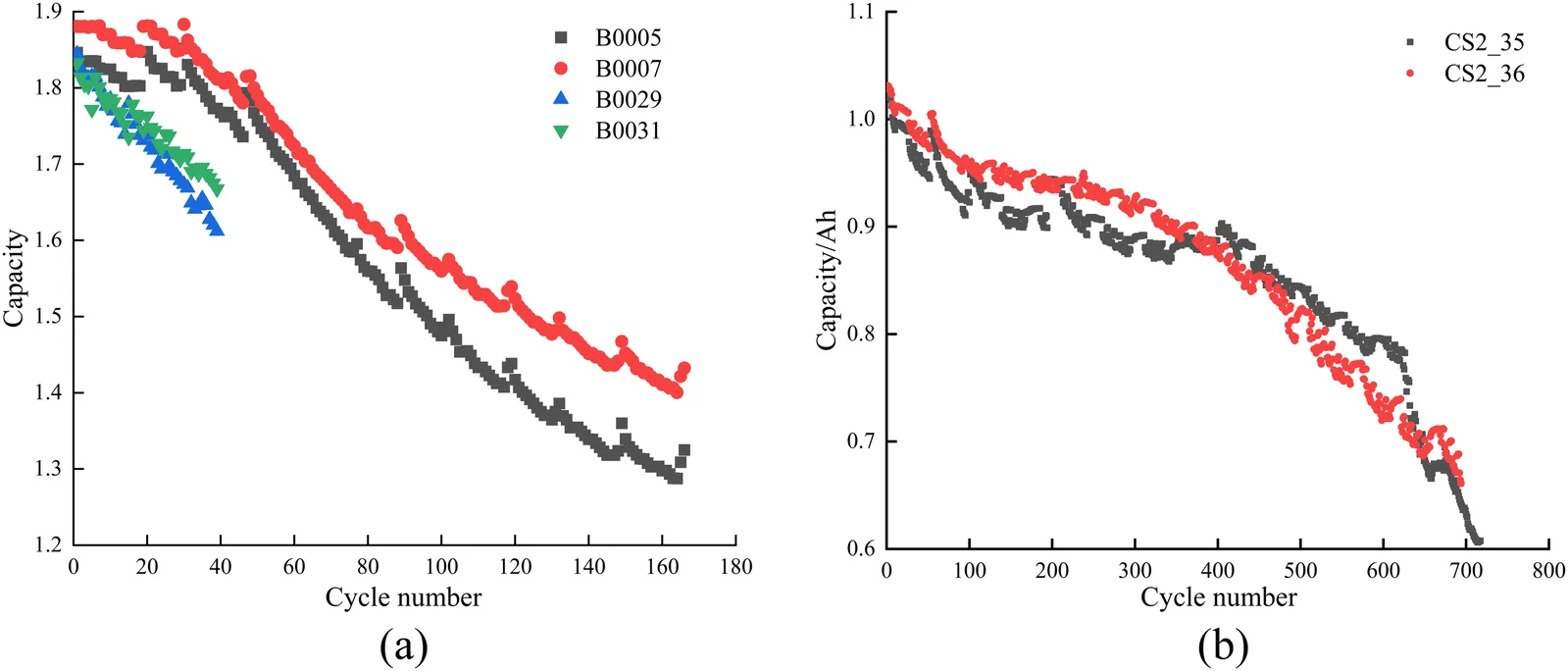
Battery capacity degradation trajectories of NASA (a) and CALCE (b).

### 2.2. Analysis and feature extraction of IC/DV curves

Incremental Capacity Analysis and Differential Voltage Analysis have been applied in the study of battery aging mechanisms [30,31]. Through the peaks and valleys in the corresponding curves, aging-related information is obtained as health features for SOH prediction.

Based on the terminal voltage-capacity data obtained in the constant-current charging stage of the battery, Savitzky-Golay filtering was applied to the IC curve and Gaussian filtering was used for the DV curve to reduce noise caused by measurement errors during the experiment.

As shown in Figs 2(a) and 3(a), the IC curves of cells B0005 and CS2_ 35 during charging at different cycle numbers are plotted against the terminal voltage. The horizontal coordinate of the x-axis is set to terminal voltage, and the vertical coordinate is incremental capacity (dQ/dV). With battery ageing, the peak height in the IC curve decreases, the position of the peak moves to higher voltages, and the area under the peak also decreases. This change is mainly due to the loss of lithium inventory (LLI): The continuous increase of the solid electrolyte interphase (SEI) layer is an irreversible consumption of cyclable lithium ions, which weakens the phase transition reaction and manifests as a decrease in peak amplitude. At the same time, a more significant electrochemical driving force needs to be applied, corresponding to an increased voltage, in order to insert fewer lithium ions; therefore, the phase transition potential rises and manifests as a peak shift to the right. Therefore, peak height, peak position and peak area can be used as health indicators to achieve precise and mechanistic interpretable battery ageing assessment. As shown in Figures 2(b) and 3(b), the differential voltage (dV/dQ) curves of cells B0005 and CS2_ 35 during charging over their entire cycle life are plotted against the terminal voltage. As the number of cycle counts increases, the peak amplitudes in the DV curve gradually decrease; as for the peak positions, they shift towards higher voltage levels; Additionally, at this time, the entire shape tends to flatten and lose distinct characteristics. This change is mainly due to the decrease in LLI and LAM within the battery: LLI reduces the number of cyclable lithium ions, weakens the phase transition reaction (reflected in a reduced peak height), and requires a higher voltage to drive the remaining reactions (manifested as a rightward peak shift); at the same time, LAM further deteriorates the electrode reaction kinetics, causing a reduction in the rate of change of the voltage. Considering that they are highly correlated with battery capacity decay, the inflection points of the charging differential voltage in the DV curve can be used as health indicators to achieve SOH forecasting.

**Fig 2:**
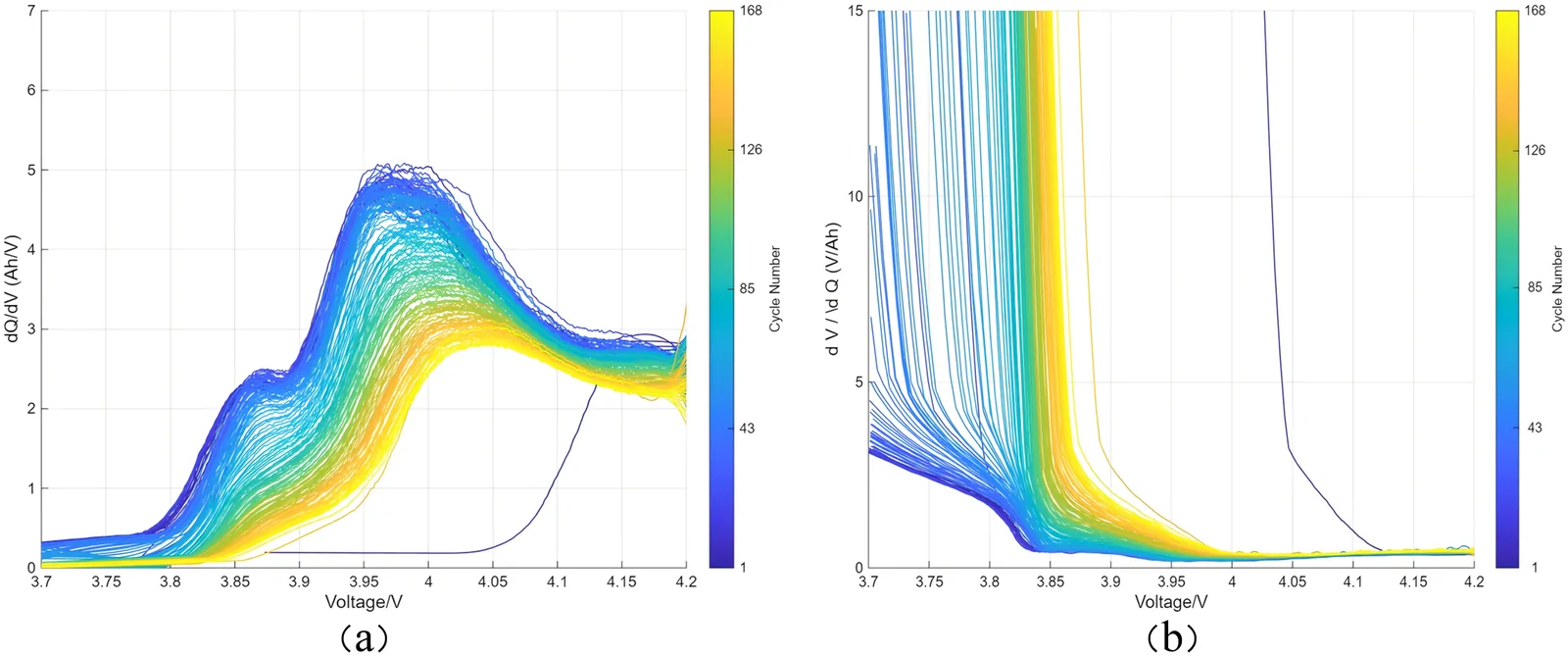
Incremental Capacity (a) and Differential Voltage (b) Curves of NASA Battery B0005.

**Fig 3:**
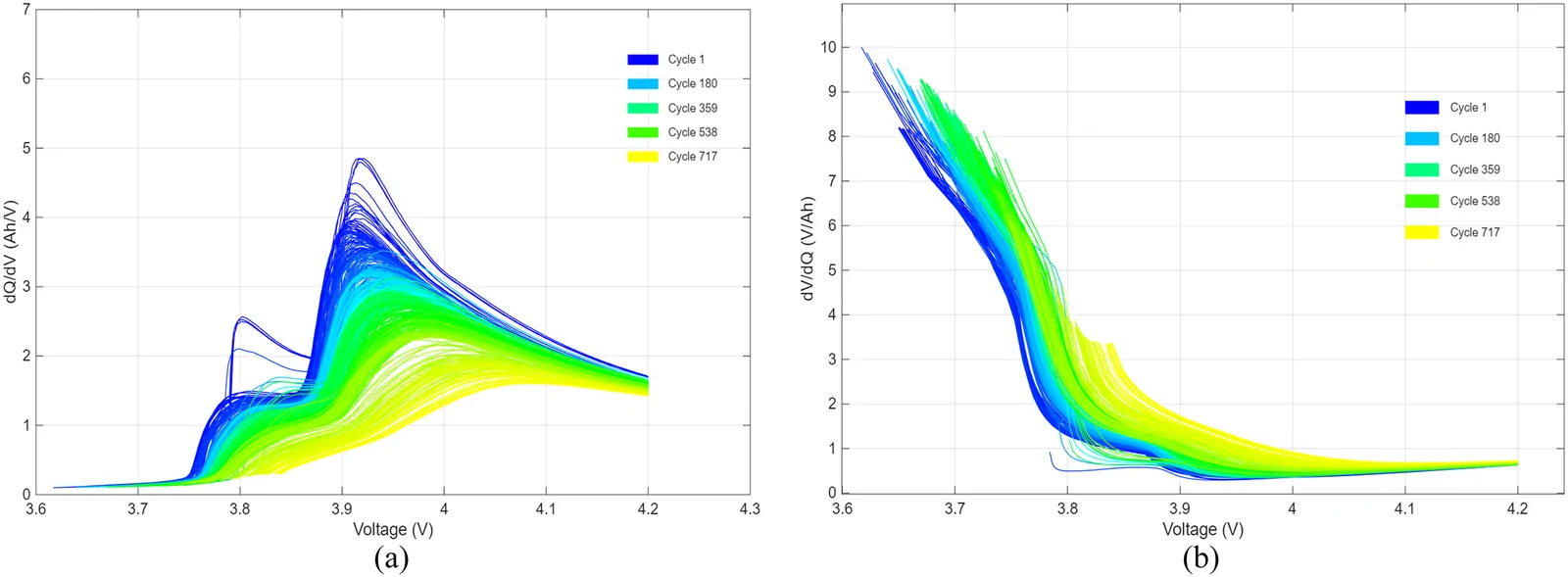
Incremental Capacity (a) and Differential Voltage (b) Curves of NASA Battery CS2_ 35.

### 2.3. Correlation analysis of health features

Based on the above IC and DV curve analyses, four health features are extracted: peak height, peak position, peak area (within the 3.7−3.9V voltage window) of the IC curve and inflection point voltage of the charging DV curve, which are represented as HF1-HF4, respectively. To what extent do two sequences that vary at different times or under various circumstances have a statistical relationship, this is called the degree of correlation. Based on Pearson’s correlation coefficient for each feature parameter in the dynamic system to determine whether it exhibits a change trend, which then evaluates battery capacity via quantified linear relationships. Compute the Pearson correlation coefficient as follows:


$$\begin{array}{c} \rho_{X,Y}=\frac{E[XY]-E[X]E[Y]}{\sqrt{E[X^{2}]-(E[X]{)}^{2}}\;\sqrt{E[Y^{2}]-(E[Y]{)}^{2}}} \end{array}$$ (1)

where *X* and *Y* denote the sequences of the feature parameter and the capacity, respectively. $\rho_{X,Y}$ denotes the correlation coefficient between the two variables; a higher value of this coefficient indicates a stronger linear relationship between them.

As shown in Table 3 (NASA dataset) and Table 4 (CALCE dataset), HF1 exhibits a strong correlation with capacity for batteries B0005 (0.9098) and B0007 (0.9731), as well as for cells in the CALCE dataset. However, its correlation noticeably weakens for B0029 (0.7785) and drops significantly lower for B0031 (0.6325). This substantial variability across different battery cells suggests that HF1 may be more sensitive to specific aging conditions, such as variations in operating temperature or discharge rates (as detailed in Table 1), leading to inconsistent performance as a health indicator. In contrast, health features HF2 to HF4 exhibit consistently high and stable correlations (absolute values > 0.81) with capacity across all four battery types. To ensure the robustness and generalization capability of our SOH estimation model, we prioritize features that demonstrate consistent reliability across diverse battery datasets over those with high but unstable correlations. Therefore, in order to improve the accuracy and robustness of SOH estimation, this study selects HF2-HF4 derived from IC and DV curves as health features for model training and testing.

**Table 3:** Pearson correlation coefficients between different features and capacity in the NASA dataset.

Battery Number	HF1	HF2	HF3	HF4
B0005	0.9098	−0.8681	0.9274	0.9082
B0007	0.9731	−0.8752	0.9389	0.9126
B0029	0.7785	−0.8136	0.9615	0.8623
B0031	0.6325	−0.8723	0.8596	0.8572

**Table 4:** Pearson correlation coefficients between different features and capacity in the CALCE dataset.

Battery Number	HF1	HF2	HF3	HF4
CS2_ 35	0.9503	−0.9815	0.9901	−0.8525
CS2_ 36	0.9654	−0.9808	0.9915	−0.9466

### 2.4. Health feature optimization

Since the HFs all have a high correlation with capacity, some degree of redundancy or information overlap among them is inevitable. To reduce the computational complexity caused by the presence of redundant and overlapped features in the high-frequency domain, KPCA is used to optimise the selected HFs. KPCA extends traditional PCA to handle nonlinear structures by first mapping the original data to a high-dimensional feature space through a kernel function, and then performing linear dimensionality reduction using PCA in that transformed space. To address the deficiency in traditional PCA that it has a weak ability to extract features when the linear correlation among sample features is low [32]. The primary steps for realising KPCA are outlined below.

Step 1: Data collection and standardization: The extracted health features are assembled into a data matrix and standardized to obtain the normalized matrix 𝐗, expressed as:


$$\begin{array}{cc} 𝐗 & ={\left[\begin{array}{cccc} x_{11} & x_{12} & \cdots & x_{1n}\\ x_{21} & x_{22} & \cdots & x_{2n}\\ \vdots & \vdots & \ddots & \vdots \\ x_{m1} & x_{m2} & \cdots & x_{mn} \end{array}\right]}^{⊤}=[x_{1}^{\prime }\;x_{2}^{\prime }\;\cdots \;x_{m}^{\prime }] \end{array}$$ (2)

where *m* represents the number of extracted health features, *n* denotes the number of battery samples, and $x_{ij}$ (with i=1,2,...,m and j=1,2,...,n) corresponds to the *j*-th sample of the *i*-th health feature.

Step 2: Kernel Matrix Construction: The Gaussian radial basis function (RBF) kernel is adopted as the nonlinear mapping kernel in KPCA. The kernel matrix 𝐊 is computed as:


$$\begin{array}{c} 𝐊=k_{RBF}({𝐱}_{k},{𝐱}_{g}) \end{array}$$ (3)

where ${𝐱}_{k}$ and ${𝐱}_{g}$ are two samples from the input matrix, and the RBF kernel function is defined as


$$\begin{array}{c} k_{RBF}(𝐮,𝐯)=\exp\left(-\frac{\left\|𝐮-𝐯\right\|}{\sigma^{2}}\right) \end{array}$$ (4)

Step 3: Kernel Matrix Centering: The kernel matrix 𝐊 is centered to yield ${𝐊}^{\prime }$, given by:


$$\begin{array}{c} {𝐊}^{\prime }=𝐊-\frac{1}{m}𝐞{𝐞}^{⊤}𝐊-𝐊\frac{1}{m}𝐞{𝐞}^{⊤}+\frac{1}{m^{2}}𝐞{𝐞}^{⊤}𝐊𝐞{𝐞}^{⊤} \end{array}$$ (5)

where 𝐞 denotes a column vector whose entries are all equal to 1.

Step 4: The original data are mapped into a high-dimensional feature space via a nonlinear mapping function $\phi ({𝐱}_{k})$, and the covariance matrix 𝐂 is subsequently computed in this transformed space.


$$\begin{array}{c} 𝐂=\frac{1}{m}\sum_{k=1}^{m}\phi (x_{k})\cdot \phi (x_{k}{)}^{⊤} \end{array}$$ (6)

Step 5: The eigenvalues λ and eigenvectors 𝐕 of the covariance matrix are computed, fulfilling the following relationship:


$$\begin{array}{c} \lambda 𝐕=𝐂𝐕 \end{array}$$ (7)

By incorporating the nonlinear mapping $\phi ({𝐱}_{k})$, Equation (6) can be reformulated as:


$$\begin{array}{c} \lambda \phi (x_{k})𝐕-\phi (x_{k})𝐂𝐕=0. \end{array}$$ (8)

By integrating Equations (6) and (7), the eigenvector 𝐕 can be derived from the following expression:


$$\begin{array}{c} 𝐕=\sum_{k=1}^{m}\alpha_{k}\phi (x_{k}). \end{array}$$ (9)

Step 6: The centered kernel matrix ${𝐊}^{\prime }$ is employed to compute the projection of the sample onto the nonlinear feature space induced by $\phi ({𝐱}_{k})$. Given that ${𝐊}^{\prime }=\langle \phi ({𝐱}_{k}),\phi ({𝐱}_{g})\rangle$ and integrating Equations (6)-(9), Equation (7) can be simplified as:


$$\begin{array}{c} m\lambda \alpha ={𝐊}^{\prime }\alpha . \end{array}$$ (10)

where α denotes the eigenvector of ${𝐊}^{\prime }$, the projection of the sample onto the nonlinear mapping ϕ(𝐱) can be formulated as:


$$\begin{array}{c} 𝐕\cdot \phi (x)=\sum_{k=1}^{m}\alpha_{k}\phi (x_{k})\phi (x)=\sum_{k=1}^{m}\alpha_{k}𝐊(x_{k},x). \end{array}$$ (11)

Step 7: The cumulative contribution rate $D_{\Sigma }$ of the kernel principal components was computed using Equation (12), and the principal components with $D_{\Sigma }\geq 90\%$ were selected as the indirect HFs for the subsequent lithium-ion battery lifetime prediction model.


$$\begin{array}{c} D_{\Sigma }=\frac{\sum_{i=1}^{k}\lambda_{i}}{\sum_{i=1}^{m}\lambda_{i}},\;(k=1,2,...,m). \end{array}$$ (12)

Tables 5 and 6 list the contribution rates of each principal component after dimensionality reduction for all batteries. For each battery, the proportion of the first principal component that accounts for more than 90% of the total variance is higher than 90%. Therefore, this study takes the first principal component obtained after dimensionality reduction as the IHF for SOH estimation.

**Table 5:** Contribution rates of principal components for the NASA dataset.

Battery Number	Principal Component 1	Principal Component 2	Principal Component 3	Principal Component 4
B0005	0.9914	0.0052	0.0031	0.0003
B0007	0.9731	0.0213	0.0034	0.0022
B0029	0.9547	0.0328	0.0093	0.0032
B0031	0.9856	0.0069	0.0040	0.0035

**Table 6:** Contribution rates of principal components for the CALCE dataset.

Battery Number	Principal Component 1	Principal Component 2	Principal Component 3	Principal Component 4
CS2_ 35	0.9832	0.0083	0.0046	0.0039
CS2_ 36	0.9652	0.0284	0.0052	0.0012

## 3. SOH estimation method

### 3.1. Transformer-LSTM model architecture

The SOH estimation model in this paper takes the indirect health feature sequence obtained through dimensionality reduction with KPCA as its input. First, the Transformer encoder is used to capture the global context information in the sequence; then the LSTM network is employed to model the time evolution of battery degradation during charge and discharge cycles.

(1) Input Preprocessing Layer: The dimensionality-reduced health feature vectors are normalized to form an input sequence of length *N*, denoted as $𝐗=[{𝐱}_{1},{𝐱}_{2},...,{𝐱}_{N}]$, which serves as the initial input to the model.
(2) Transformer Encoder Layer: This layer is composed of multiple modules, including position encoding, multi-head self-attention and feedforward neural network, which are designed to capture the long-distance degradation rules contained in the health feature sequence.

$$\begin{array}{c} P_{E}(pos,2i)=\sin\left(\frac{pos}{10000^{2i/d}}\right) \end{array}$$ (13)

$$\begin{array}{c} P_{E}(pos,2i+1)=\cos\left(\frac{pos}{10000^{2i/d}}\right) \end{array}$$ (14)

where $P_{E}$ represents the positional encoding, pos indicates the position of the element within the sequence, *d* is the dimensionality of the positional encoding, and *i* denotes the index along the feature dimension.

Subsequently, the multi-head self-attention mechanism calculates the correlation weight matrix of health features at different time steps. The outputs of all attention heads are first concatenated and then combined using a learnable linear transformation. Denoting the input to the self-attention module as the matrix 𝐗, the output is obtained by computing the query matrix 𝐐, key matrix 𝐊, and value matrix 𝐕 as follows:


$$\begin{array}{c} \left\{ \begin{array}{c} 𝐐=𝐗{𝐖}^{Q}\\ 𝐊=𝐗{𝐖}^{K}\\ 𝐕=𝐗{𝐖}^{V} \end{array}\right. \end{array}$$ (15)

$$\begin{array}{c} Attention(Q,K,V)=softmax\left(\frac{QK^{⊤}}{\sqrt{d_{k}}}\right)V \end{array}$$ (16)

where Attention(Q,K,V) denotes the attention scores, ${𝐖}^{Q}$, ${𝐖}^{K}$, and ${𝐖}^{V}$ are trainable weight matrices in the model, and $d_{k}$ denotes the dimensionality of the key vectors.

Subsequently, a feed-forward neural network is utilized to strengthen the model’s ability to capture nonlinear relationships. Both the attention module and the feed-forward module are followed by residual connections and layer normalization:


$$\begin{array}{c} Output=LayerNorm(Sublayer(Z)+Z) \end{array}$$ (17)

where Output denotes the output of the current module, which is passed as input to the subsequent layer, and *Z* represents the input to the current sub-layer (the feature sequence generated by the preceding layer).

(3) LSTM Temporal Modeling Layer: The high-dimensional representation sequence output by the Transformer encoder, denoted as 𝐙, is fed into the LSTM layer. By adaptively modulating information flow through its three gating mechanisms-the forget gate, input gate, and output gate-the LSTM effectively alleviates the long-term dependency problem. Its state update equations are as follows:

$$\begin{array}{cc} F_{t} & =\sigma (W_{f}\cdot [X_{t},H_{t-1}]+b_{f}) \end{array}$$ (18)

$$\begin{array}{cc} I_{t} & =\sigma (W_{i}\cdot [X_{t},H_{t-1}]+b_{i}) \end{array}$$ (19)

$$\begin{array}{cc} O_{t} & =\sigma (W_{o}\cdot [X_{t},H_{t-1}]+b_{o}) \end{array}$$ (20)

$$\begin{array}{cc} {\tilde{C}}_{t} & =\tanh(W_{c}\cdot [X_{t},H_{t-1}]+b_{c}) \end{array}$$ (21)

$$\begin{array}{cc} C_{t} & =F_{t}\cdot C_{t-1}+I_{t}\cdot {\tilde{C}}_{t} \end{array}$$ (22)

$$\begin{array}{cc} H_{t} & =O_{t}\cdot \tanh(C_{t}) \end{array}$$ (23)

where $F_{t}$, $I_{t}$, and $O_{t}$ represent the forget gate, input gate, and output gate, respectively; ${\tilde{C}}_{t}$ denotes the candidate memory cell; $C_{t}$ is the updated memory cell; $H_{t}$ represents the hidden state at time step *t*; σ and tanh are the sigmoid and hyperbolic tangent activation functions, respectively; $X_{t}$ is the input at time step *t*; $H_{t-1}$ is the hidden state from the previous time step; 𝐖 denotes the weight matrices; and 𝐛 deno*t*es the bias vectors.

(4) Output Prediction Layer: The sequence of hidden states produced by the LSTM, denoted as $𝐇=[{𝐡}_{1},...,{𝐡}_{T}]$, is mapped to a scalar SOH prediction value through a fully connected layer with one neuron and linear activation:

$$\begin{array}{c} {\hat{y}}_{t}={𝐰}^{⊤}{𝐡}_{t}+b \end{array}$$ (24)

where ${\hat{y}}_{t}$ denotes the estimated SOH at the *t*-th cycle.

### 3.2. Newton-Raphson-based optimization algorithm

In 2024, the research team led by S. Ravichandran developed a novel swarm intelligence algorithm called the Newton-Raphson-based Optimization algorithm. The algorithm integrates two core mechanisms: first, it introduces the Newton-Raphson Search Rule (NRSR), which constructs a gradient-responsive model to expand the exploration range of the solution space, thereby significantly enhancing global optimization efficiency; second, it incorporates a parameter perturbation escape mechanism, termed the Trap Avoidance Operator (TAO), which employs a probabilistic update strategy to break the correlation among individual solutions, effectively addressing the issue of conventional optimization algorithms becoming trapped in local optima. Within the scope of this research, the NRBO algorithm is employed to automatically tune the key hyperparameters of the hybrid Transformer-LSTM neural network, such as the number of attention heads, the learning rate, and the regularization coefficient. The detailed implementation procedure is as follows:

(1) Newton-Raphson Search Rule

Use the NRSR rule to explore new solutions. For the *n*-th individual at the *N*-th iteration, its position is updated via NRSR, and the updated position is expressed as follows:


$$\begin{array}{c} x_{n}^{N+1}=r_{1}\left[r_{1}X1_{n}^{N}+(1-r_{1})X2_{n}^{N}\right]+(1-r_{1})X3_{n}^{N} \end{array}$$ (25)

The expressions for $X1_{n}^{N}$, $X2_{n}^{N}$, and $X3_{n}^{N}$ are as follows:


$$\begin{array}{c} X1_{n}^{N}=x_{n}^{N}-Nt+Rh \end{array}$$ (26)

$$\begin{array}{c} X2_{n}^{N}=x_{b}-Nt+Rh \end{array}$$ (27)

$$\begin{array}{c} X3_{n}^{N}=x_{n}^{N}-\delta (X2_{n}^{N}-X1_{n}^{N}) \end{array}$$ (28)

The expressions for *Nt*, *Rh*, and δ are as follows:


$$\begin{array}{c} N_{t}=randn\times \frac{(y_{c}-y_{a})\Delta x}{2(y_{c}+y_{d}-2x_{n}^{N})} \end{array}$$ (29)

$$\begin{array}{c} Rh=c(x_{b}-x_{n}^{N})+g(x_{a1}^{N}-x_{a2}^{N}) \end{array}$$ (30)

$$\begin{array}{c} \delta ={\left(1-\frac{2N}{N_{\max}}\right)}^{5} \end{array}$$ (31)

where: randn refers to a random number drawn from a standard normal distribution with mean 0 and variance 1; the value of δ varies within the interval [−1,1]; and Δx represents the perturbation magnitude.

In the optimisation of the Transformer-LSTM model, NRSR helps the algorithm effectively search the combined space of learning rate and network architecture to avoid suboptimal configurations such as too small a learning rate causing slow convergence or even no convergence at all.

(2) Trap Avoidance Operator

When the algorithm determines that it has reached a local optimum, the TAO addresses this problem by combining the current position with the best-known position to produce an improved solution ${𝐱}_{TAO}^{N}$. The expression to generate this new solution is as follows:


$$\begin{array}{c} x_{n}^{N+1}=x_{TAO}^{N},\;\text{if }rand<DF \end{array}$$ (32)

$$\begin{array}{c} x_{TAO}^{N}=\left\{ \begin{array}{cc} x_{n}^{N}+\theta_{1}(\mu_{1}x_{b}-\mu_{2}x_{n}^{N})+\theta_{2}\delta [\mu_{1}Mean(x^{N}-\mu_{2}x_{n}^{N})], & \text{if }\mu_{1}<0.5\\ x_{b}+\theta_{1}(\mu_{1}x_{b}-\mu_{2}x_{n}^{N})+\theta_{2}\delta [\mu_{1}Mean(x^{N}-\mu_{2}x_{n}^{N})], & \text{if }\mu_{1}\geq 0.5 \end{array}\right. \end{array}$$ (33)

where: $\theta_{1}$ and $\theta_{2}$ are randomly generated values varying within [−1,1] and [−0.5,0.5], respectively; Mean(·) denotes the averaging function; $\mu_{1}$ and $\mu_{2}$ are randomly generated values used to control population diversity.

During Transformer-LSTM training, if multiple candidate hyperparameter configurations lead to a plateau in validation loss-indicating entrapment in a local optimum-the TAO generates new configurations with enhanced generalization potential by fusing the global best solution $x_{b}$ and the population centroid $Mean(x^{N})$. For example, it may increase the number of hidden units to expand model capacity while simultaneously adjusting the regularization strength to prevent overfitting.

Fig 4 illustrates the flow of the NRBO algorithm.

**Fig 4:**
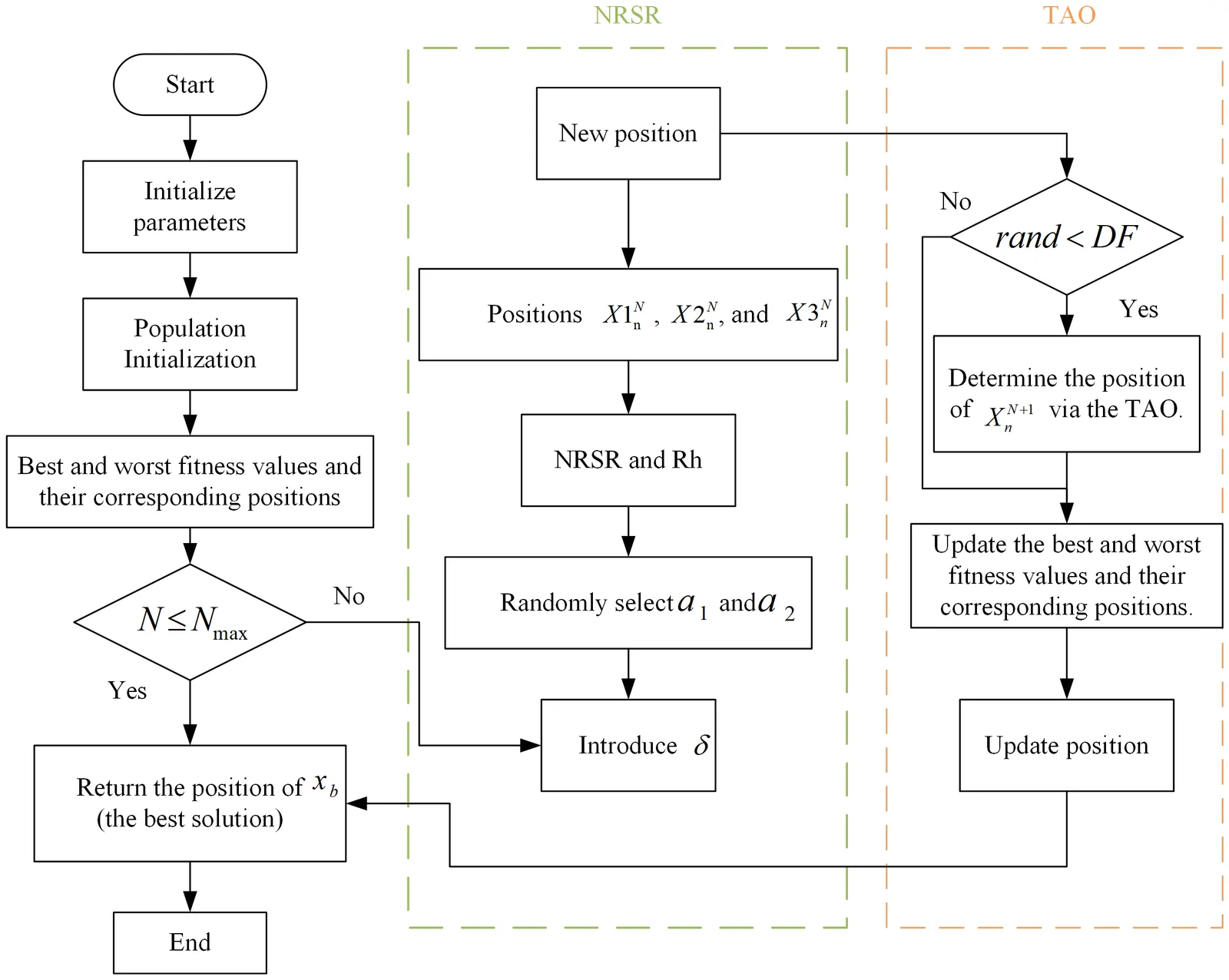
NRBO Algorithm Flowchart.

### 3.3. SOH estimation procedure

This study presents a SOH estimation framework that combines feature engineering with intelligent optimization. First, a group of novel battery degradation indicators is derived from the filtered incremental capacity and differential voltage curves. Nonlinear dimensionality reduction is then performed in the high-dimensional feature space using KPCA to obtain the key components that effectively characterize battery degradation. Based on different data partitioning strategies, the preprocessed dataset is split into training and test subsets. During the model training phase, the NRBO algorithm is employed to globally optimize three critical hyperparameters of the Transformer-LSTM model: the number of attention heads, the learning rate, and the L2 regularization coefficient, thereby establishing an adaptive battery degradation prediction model. Finally, the performance of the proposed NRBO-Transformer-LSTM framework for SOH estimation is evaluated on both the NASA and CALCE battery datasets. The NRBO algorithm is compared against other optimization algorithms to highlight its advantages, and both longitudinal and cross-sectional comparative experiments are conducted to comprehensively validate the superiority of the proposed method.

## 4. Experimental evaluation

### 4.1. Assessment criteria

To quantitatively evaluate the accuracy of the model prediction from multiple perspectives, this study adopts two key metrics for a comprehensive evaluation: MAE, which measures the average absolute deviation between predicted and true values, and RMSE, which places greater emphasis on the impact of larger errors on overall performance.


$$\begin{array}{c} f_{MAE}=\frac{1}{n}\sum_{i=1}^{n}|y_{i}-\bar{y}| \end{array}$$ (34)

$$\begin{array}{c} f_{RMSE}=\sqrt{\frac{1}{n}\sum_{i=1}^{n}(y_{i}-\bar{y}{)}^{2}} \end{array}$$ (35)

where $y_{i}$ denotes the true SOH value, $\bar{y}$ represents the predicted SOH value, and *n* is the length of the data.

### 4.2. Comparison of optimization algorithms

To assess the precision and robustness of the proposed battery age prediction model, 70% of the cycles from each of the four batteries will be used to train the model, while the remaining 30% will be reserved for testing. For example, for batteries B0005 and B0007, cycles 1–116 are the training set, and cycles 117–166 are the test set; For batteries B0029 and B0031, the first 27 cycles are used as the training set, and cycles 28–39 are used as the test set.

As shown in Figs 5 and 6 and Tables 7, among them, the NRBO algorithm is always able to obtain a more accurate trajectory of evolution of SOH, and its predicted SOH curve is closer to the actual value than that of GWO and WOA. Quantitative error metrics show that both the MAE and RMSE of NRBO are below 1%. In addition, the SOH prediction curves of GWO and WOA have more fluctuations. Although they can indicate the general deterioration trend of the battery, due to their higher prediction errors and a local optimum tendency, their accuracy is limited. Nonlinear degradation phenomena, such as local capacity variations and capacity recovery, cannot be accurately captured by the fixed-length search and random-walk mechanisms of GWO; However, using the spiral-search and random-walk mechanisms of WOA is more likely to obtain the true degradation rules and therefore provide prediction results closer to the actual SOH. Nevertheless, NRBO has demonstrated the most stable and accurate performance overall.

**Table 7:** Comparison of SOH estimation error results in optimization algorithm comparative experiments.

Battery	Method	MAE	RMSE
B0005	GWO	0.0137	0.0164
	WOA	0.0110	0.0126
	NRBO	0.0059	0.0082
B0007	GWO	0.0217	0.0254
	WOA	0.0111	0.0125
	NRBO	0.0053	0.0079
B0029	GWO	0.0197	0.0232
	WOA	0.0112	0.0143
	NRBO	0.0084	0.0091
B0031	GWO	0.0136	0.0156
	WOA	0.0093	0.0122
	NRBO	0.0072	0.0086
CS2_ 35	GWO	0.0163	0.0189
	WOA	0.0133	0.0163
	NRBO	0.0071	0.0089
CS2_ 36	GWO	0.0164	0.0186
	WOA	0.0127	0.0149
	NRBO	0.0066	0.0087

**Fig 5:**
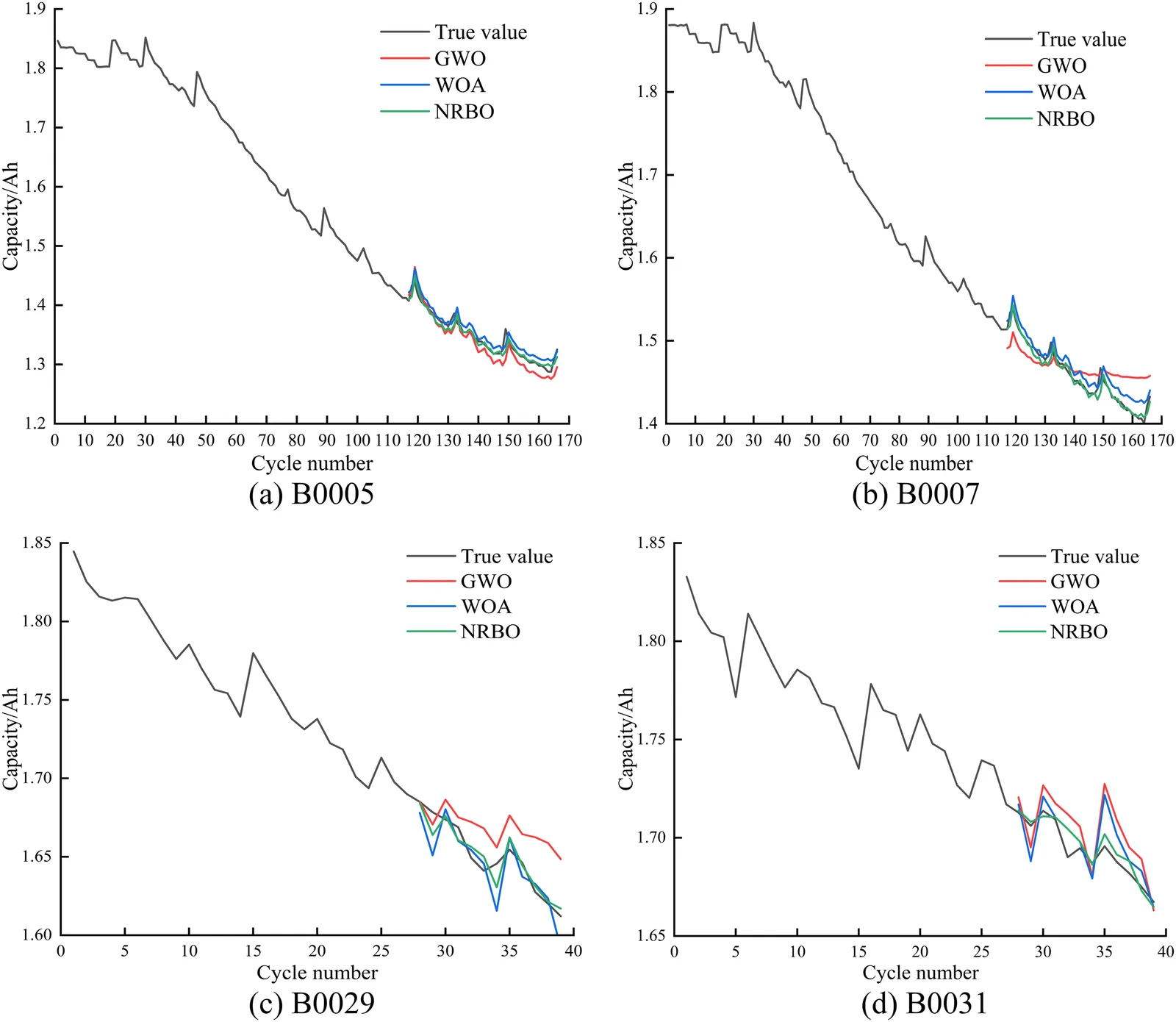
SOH Estimation Results on NASA Dataset in Optimization Algorithm Comparative Experiments.

**Fig 6:**
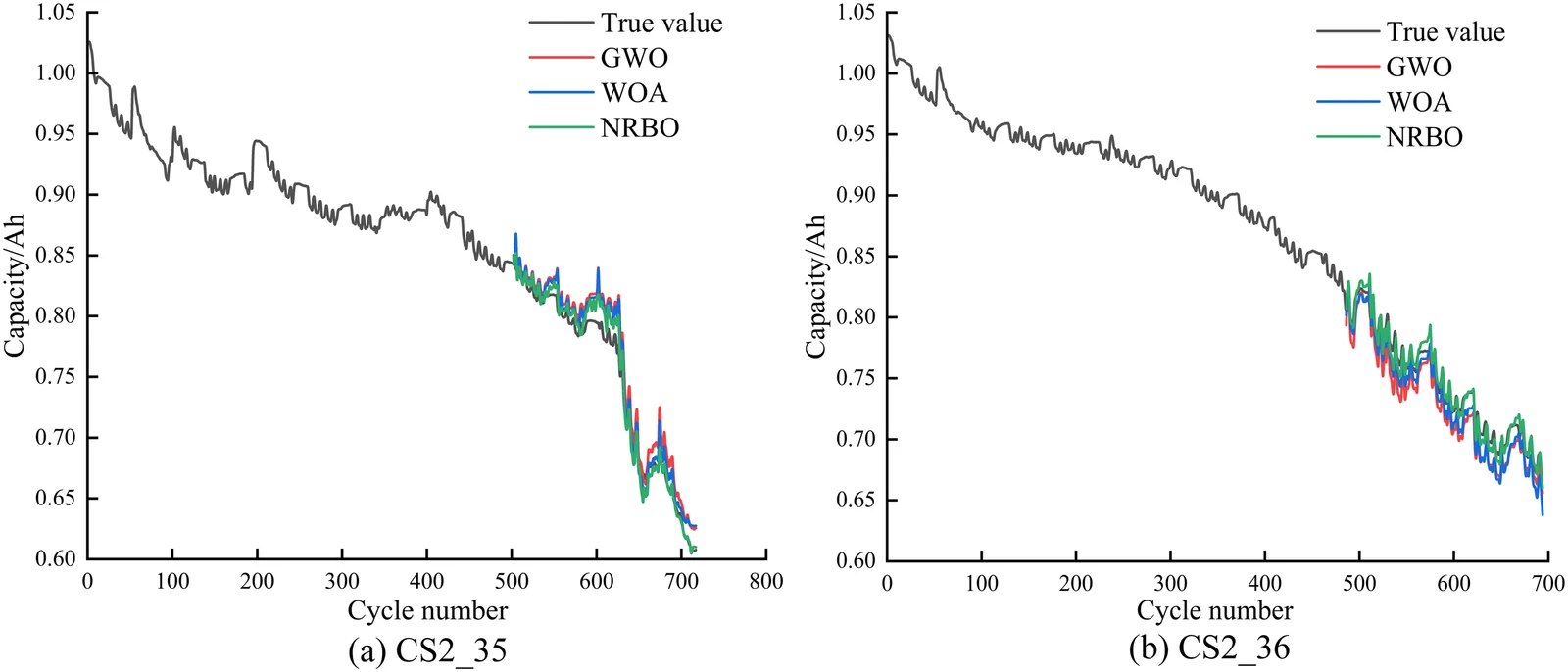
SOH Estimation Results on CALCE Dataset in Optimization Algorithm Comparative Experiments.

### 4.3. Longitudinal comparative experiment

The proposed NRBO-Transformer-LSTM model is benchmarked against three baseline architectures: LSTM, Transformer, and Transformer-LSTM. The prediction results are visualized in Fig 7 and Fig 8. In Table 8, M1-M4 denote the three baseline models and the proposed NRBO-Transformer-LSTM model, respectively, for comparative analysis. As shown in Table 8, M4 consistently achieves the best performance across all test samples, yielding the lowest MAE and RMSE values among all methods. The RMSE values for the six batteries are 0.0082, 0.0079, 0.0091, 0.0086, 0.0089 and 0.0087, respectively-each lower than those achieved by other existing prediction models-highlighting the superior SOH estimation performance of the NRBO-Transformer-LSTM model.

**Table 8:** Comparison of SOH estimation error results in longitudinal comparative experiments.

Battery	Method	MAE	RMSE
B0005	M1	0.0216	0.0229
	M2	0.0150	0.0168
	M3	0.0115	0.0153
	M4	0.0059	0.0082
B0007	M1	0.0210	0.0225
	M2	0.0142	0.0165
	M3	0.0084	0.0106
	M4	0.0053	0.0079
B0029	M1	0.0171	0.0198
	M2	0.0121	0.0146
	M3	0.0107	0.0129
	M4	0.0084	0.0091
B0031	M1	0.0198	0.0219
	M2	0.0138	0.0162
	M3	0.0128	0.0148
	M4	0.0072	0.0086
CS2_ 35	M1	0.0223	0.0259
	M2	0.0213	0.0251
	M3	0.0179	0.0210
	M4	0.0071	0.0089
CS2_ 36	M1	0.0252	0.0287
	M2	0.0229	0.0269
	M3	0.0147	0.0173
	M4	0.0066	0.0087

**Fig 7:**
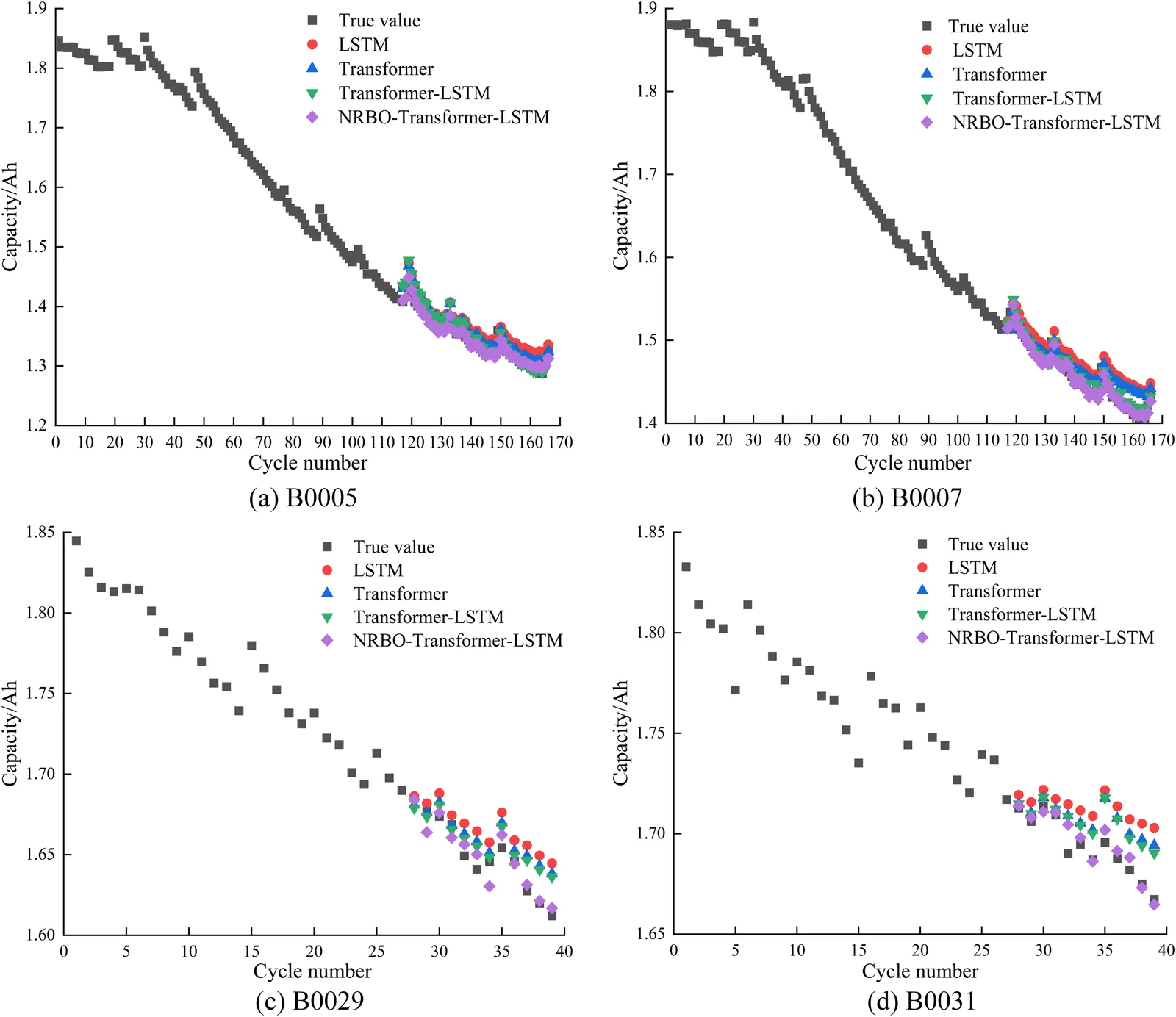
SOH Estimation Results on NASA Dataset in Longitudinal Comparative Experiment.

**Fig 8:**
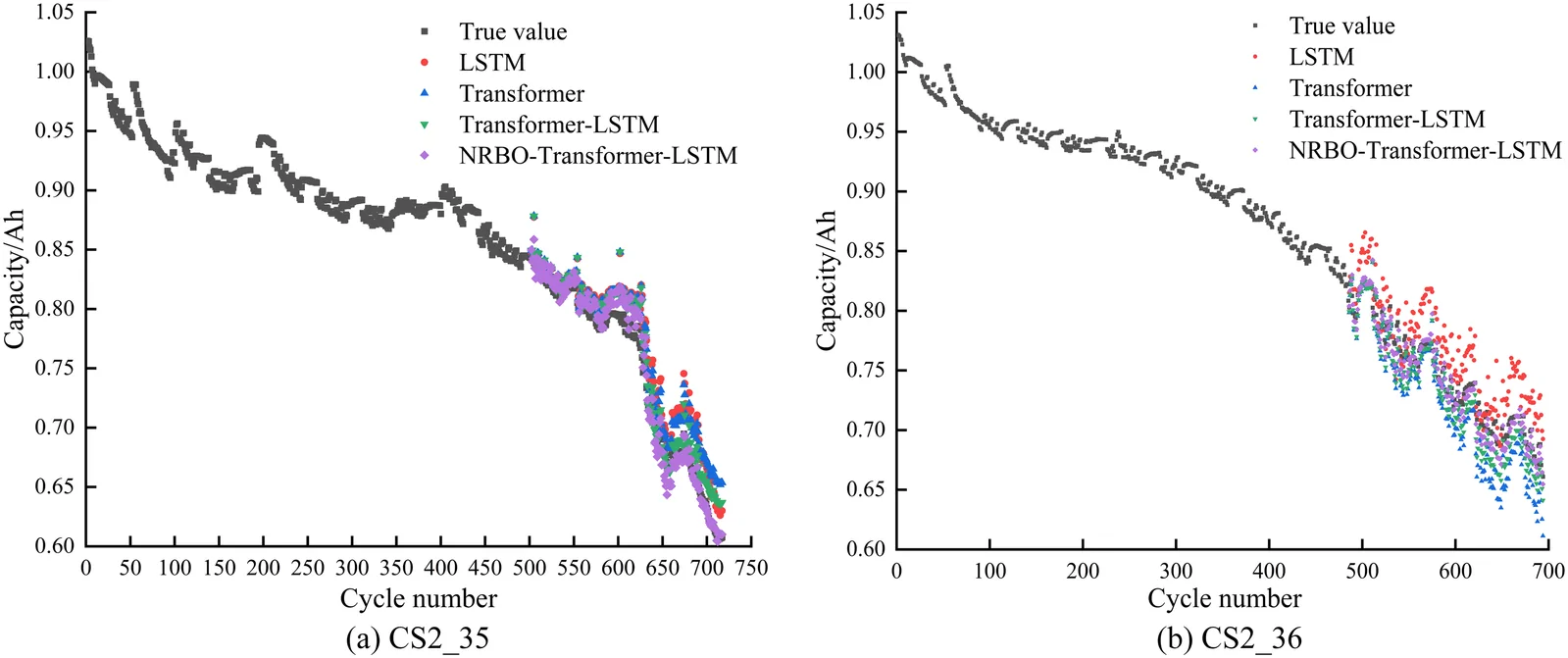
SOH Estimation Results on CALCE Dataset in Longitudinal Comparative Experiment.

### 4.4. Cross-sectional comparative experiment

To comprehensively validate the effectiveness and superiority of the proposed model, a cross-sectional comparative experiment was conducted on six batteries, that is, B0005, B0006, B0029, B0031, CS2_ 35 and CS2_ 36. And four methods are used for SOH estimation, that is, BP, GPR, ELM, and the proposed NRBO-Transformer-LSTM. These methods are denoted as N1 to N4, respectively. As shown in Fig 9, Fig 10 and Table 9, the prediction results demonstrate that, compared to the other three approaches, the proposed method (N4) achieves significantly smaller errors and its predicted values are much closer to the ground truth, thereby fully confirming the superior performance of the NRBO-Transformer-LSTM framework.

**Table 9:** Comparison of SOH estimation error results in cross-sectional comparative experiment.

Battery	Method	MAE	RMSE
B0005	N1	0.0145	0.0179
	N2	0.0151	0.0185
	N3	0.0236	0.0257
	N4	0.0059	0.0082
B0007	N1	0.0127	0.0150
	N2	0.0249	0:0275
	N3	0.0095	0.0120
	N4	0.0053	0.0079
B0029	N1	0.0192	0.0223
	N2	0.0202	0.0231
	N3	0.0143	0.0169
	N4	0.0084	0.0091
B0031	N1	0.0196	0.0216
	N2	0.0157	0.0172
	N3	0.0165	0.0184
	N4	0.0072	0.0086
CS2_ 35	N1	0.0351	0.0457
	N2	0.0432	0.0491
	N3	0.0313	0.0376
	N4	0.0071	0.0089
CS2_ 36	N1	0.0191	0.0245
	N2	0.0324	0.0336
	N3	0.0275	0.0314
	N4	0.0066	0.0087

**Fig 9:**
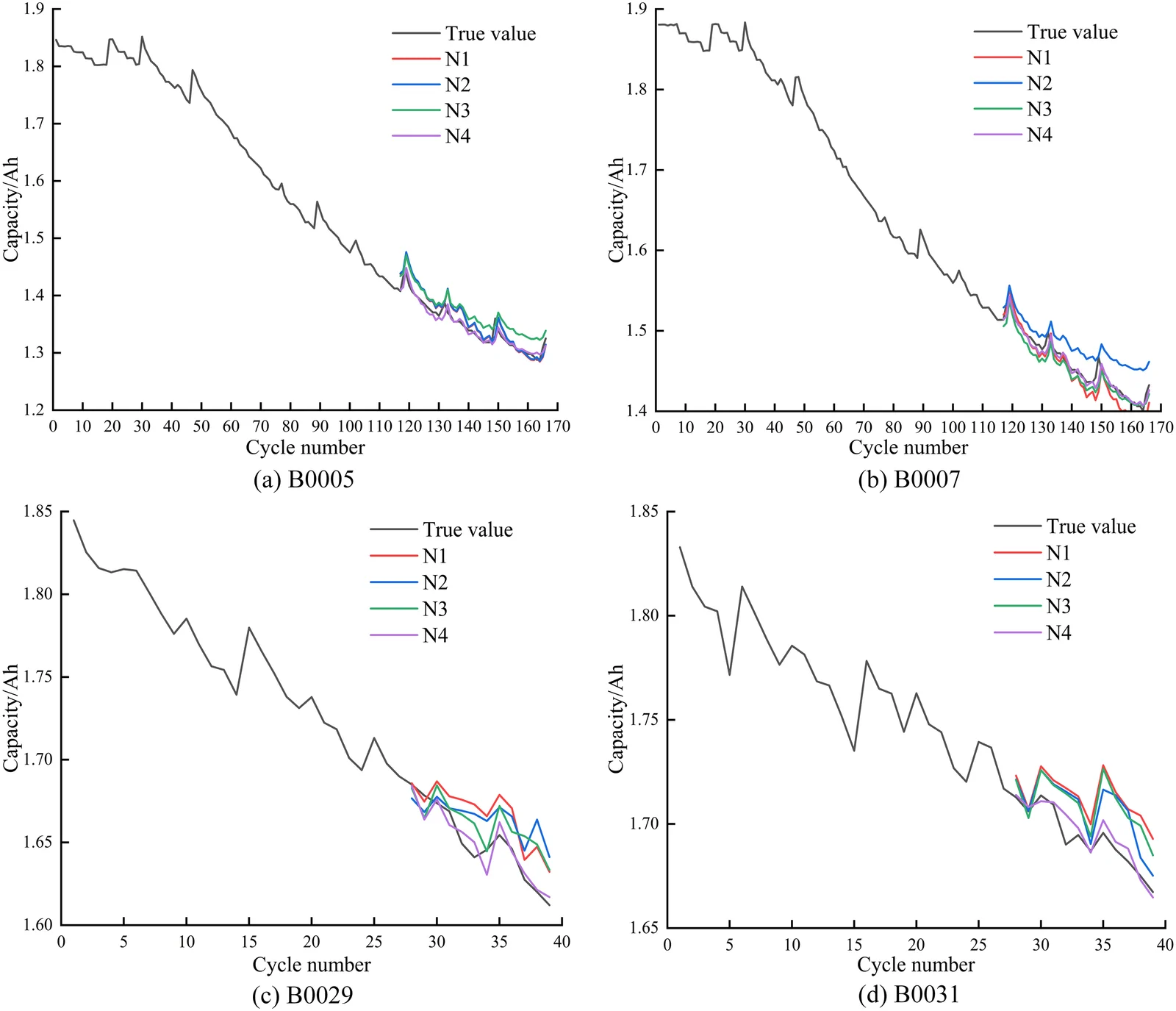
SOH Estimation Results on NASA Dataset in Cross-sectional Comparative Experiment.

**Fig 10:**
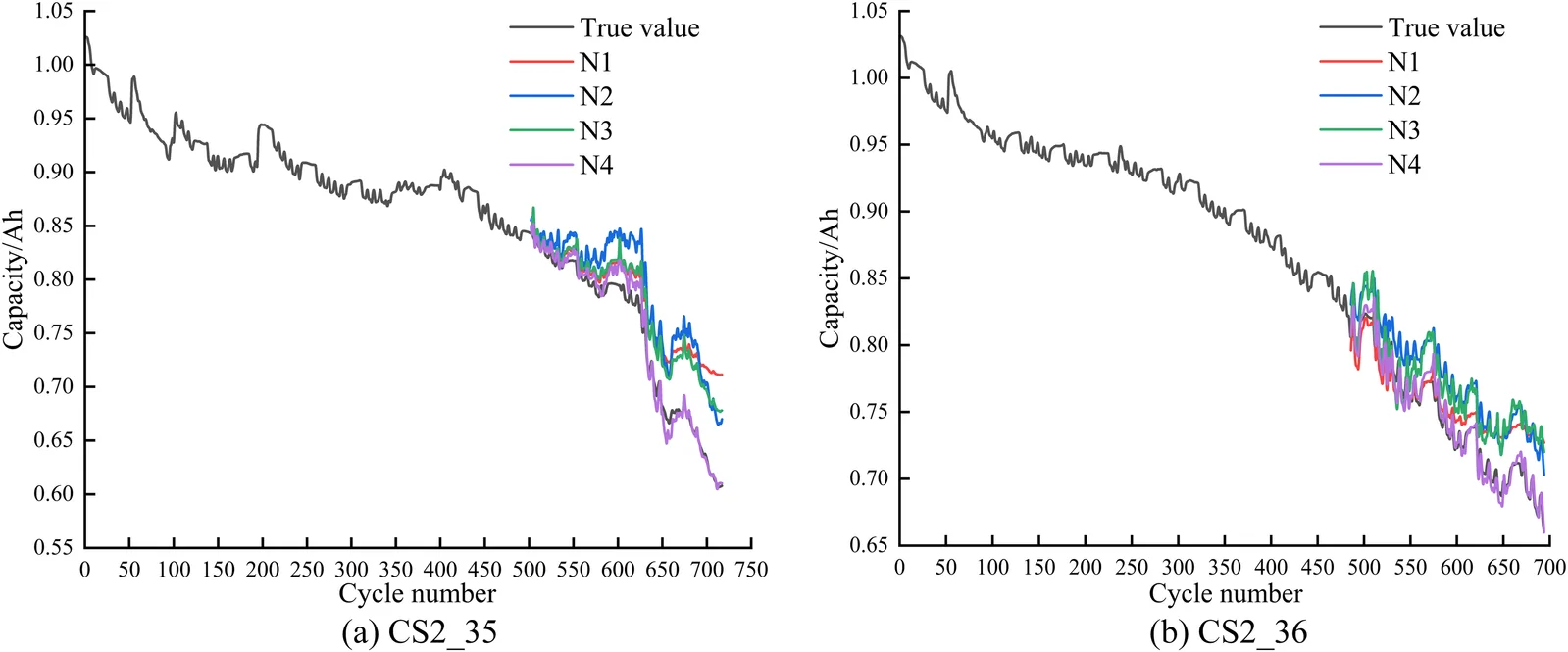
SOH Estimation Results on CALCE Dataset in Cross-sectional Comparative Experiment.

### 4.5. Computational efficiency analysis

In practical applications, a prediction method must be evaluated not only by its accuracy but also by its computational time. To further verify the superiority of the proposed NRBO-Transformer-LSTM method, we compare it with GWO and WOA in terms of computational efficiency. Table 10 records the computational time over 10 optimization iterations. It can be observed that GWO exhibits significantly lower computational time than WOA due to its fast convergence characteristic; however, this premature convergence makes it more prone to getting trapped in local optima, resulting in larger estimation errors. In contrast, NRBO-leveraging its dual mechanisms of NRSR (Novel Ranking-based Selection Rule) and TAO (Trap Avoidance Operator)-effectively escapes local optima and accelerates convergence using gradient information, thereby achieving lower computational time. This further demonstrates that the proposed model can deliver more efficient SOH estimation results.

**Table 10:** Computational time (in seconds) of different optimization algorithms for 10 iterations.

Training Set	Test Set	Optimization Algorithm	Computation Time / s
70% of B0005	30% of B0005	GWO	42
70% of B0005	30% of B0005	WOA	36
70% of B0005	30% of B0005	NRBO	17
70% of B0007	30% of B0007	GWO	45
70% of B0007	30% of B0007	WOA	34
70% of B0007	30% of B0007	NRBO	16
70% of B0029	30% of B0029	GWO	34
70% of B0029	30% of B0029	WOA	22
70% of B0029	30% of B0029	NRBO	11
70% of B0031	30% of B0031	GWO	35
70% of B0031	30% of B0031	WOA	26
70% of B0031	30% of B0031	NRBO	14
70% of CS2_ 35	30% of CS2_ 35	GWO	52
70% of CS2_ 35	30% of CS2_ 35	WOA	43
70% of CS2_ 35	30% of CS2_ 35	NRBO	21
70% of CS2_ 36	30% of CS2_ 36	GWO	54
70% of CS2_ 36	30% of CS2_ 36	WOA	37
70% of CS2_ 36	30% of CS2_ 36	NRBO	25

## 5. Discussion

The hybrid deep learning framework proposed in this study-integrating electrochemical mechanism-derived features with intelligent optimization-achieves high accuracy and reliability in lithium-ion battery SOH estimation. Its core strength lies in combining the flexibility of data-driven modeling with the physical constraints of battery aging mechanisms.

HF2-HF4 extracted from IC curves directly reflect fundamental degradation processes: shifts in the anode peak indicate LLI, while cathode changes correlate with LAM. This physics-informed feature engineering embeds domain knowledge into the model, guiding it to learn true degradation patterns rather than superficial correlations. KPCA further refines these into compact, noise-robust IHF.

To capture both long-term trends and short-term fluctuations in aging dynamics, we employ a Transformer-LSTM hybrid architecture. The Transformer models global degradation via long-range attention across the full cycle life, while the LSTM captures local transient effects caused by operational variations such as charge/discharge rate and temperature. Their synergy enables accurate reconstruction of complex, nonlinear SOH trajectories. Optimal performance is ensured by NRBO, which efficiently tunes hyperparameters to avoid local minima and enhance prediction smoothness. Unlike pure black-box models, the proposed SOH estimation scheme improves interpretability and generalization through embedded physics; unlike purely mechanistic models, it retains strong adaptability via data-driven learning. This “gray-box” paradigm-where data informs physics and physics guides data—offers a robust, deployable solution for next-generation battery management systems that balances accuracy with physical fidelity.

## 6. Conclusion

To achieve high-accuracy SOH prediction of lithium-ion batteries, this paper builds up a feature-sifting based analysis framework. Building upon these processed features, an enhanced NRBO-Transformer-LSTM model is developed for SOH forecasting. This architecture leverages the Transformer’s attention mechanism and combines it with LSTM’s capacity to model temporal dependencies. The model effectively incorporates backward contextual information and emphasizes critical features throughout the sequence. The proposed SOH prediction scheme was validated on the NASA and CALCE battery dataset, with the NRBO algorithm compared against other optimization algorithms to highlight its superiority. Both longitudinal and cross-sectional comparative experiments were designed to demonstrate the superiority of the proposed method. Experimental results show that, with a 70% training set, the proposed method achieves a maximum MAE of 0.0084 and a RMSE of 0.0091, significantly outperforming other benchmark approaches and demonstrating superior performance in both accuracy and robustness. These results confirm that the framework achieves not only high prediction accuracy but also strong robustness, offering a promising solution for reliable SOH estimation in practical battery management systems.
