# Supplementary material for: Data-driven and feature-sifting based state-of-health estimation for li-ion batteries using KPCA and NRBO-optimized transformer-LSTM hybrid model
Source: PLoS One. 2026 Oct 5;21(10):e0359015. doi: 10.1371/journal.pone.0359015 (PMC13638052; doi:10.1371/journal.pone.0359015)
Supplement: Supplementary file 1 [file pone.0359015.s001.docx]

1. According to the data in Table 2, the Pearson coefficients of HF1 (peak height of the IC curve) for batteries B0005 and B0007 reached 0.9098 and 0.9731 respectively. In many literature, a coefficient above 0.9 has been regarded as extremely strong correlation. The author only excluded HF1 in the text based on the reason of "comparatively lower", which is not sufficient. Please provide a detailed explanation as to whether the exclusion of HF1 is because of its volatility across different batteries (such as B0031 with only 0.6325) affecting the generalization ability of the model. 2. In the experimental evaluation, the author only used the NASA lithium-ion battery cycling dataset and did not conduct cross-validation across datasets or individual batteries, making it difficult to fully prove the generalization ability of the model. It is recommended to add other datasets or conduct hold-out experiments with the same dataset. 3. For the experiments of batteries B0029 and B0031, the sample sizes of the training and test sets for these two batteries are insufficient. Moreover, the Transformer encoder and LSTM network both contain complex gating mechanisms and multi-head attention mechanisms, with a large number of parameters. Training such a complex hybrid deep learning model on a training set with only 27 time steps/samples has a very high risk of overfitting. The author needs to provide a detailed explanation on how to ensure the generalization ability of the model under such a small sample size, or explore why the overall life cycle data of these two batteries is so short. 4. In the introduction section, the author correctly pointed out that some existing deep learning models are limited in real-time deployment in resource-constrained battery management systems (BMS) due to high computational costs. However, the method proposed in this paper sequentially combines KPCA dimensionality reduction, Transformer encoder, LSTM time modeling, and NRBO hyperparameter optimization algorithms. The text completely lacks a quantitative assessment of the computational complexity or inference time of the proposed model. 5. In Table 3 (Table 3), the first column lacks a header name. Based on the context data, this list header should be named "Battery". Please complete it. Additionally, the format and grammar of the article still need to be further optimized.
