# Supplementary material for: Data-driven and feature-sifting based state-of-health estimation for li-ion batteries using KPCA and NRBO-optimized transformer-LSTM hybrid model
Source: PLoS One. 2026 Oct 5;21(10):e0359015. doi: 10.1371/journal.pone.0359015 (PMC13638052; doi:10.1371/journal.pone.0359015)
Supplement: Supplementary file 2 [file pone.0359015.s003.docx]

**Original Manuscript ID:** PONE-D-26-13461

**Original Article Title: Data-Driven and Feature-Sifting Based State-of-Health Estimation for Li-Ion Batteries Using KPCA and NRBO-Optimized Transformer-LSTM Hybrid Model**

**To:** PLOS ONE Editor

**Re:** Response to reviewers

Dear Editor,

Thank you very much for giving us the opportunity to resubmit our manuscript and revise it in response to reviewers’ comments. We sincerely appreciate the time and effort that you and reviewers have devoted to evaluating our work.

In this revision, we have carefully revised the manuscript according to the reviewers’ suggestions and have prepared a point-by-point response to each comment. For clarity, we are submitting the following files:
(a) a detailed response-to-reviewers document containing our point-by-point replies;
(b) a highlighted PDF showing all revisions made in the manuscript; and
(c) a clean revised manuscript without highlights.

We hope that the revised manuscript and the responses address the concerns raised by reviewers. Thank you again for your consideration.

Best regards,

Dr. Xi Chen

China Three Gorges University

eene_chenxi@ctgu.edu.cn

**Reviewer#1, Concern # 1:** This research work employs incremental capacity analysis and differential voltage analysis to extract health features, but these methods are quite common.

**Author response:** We would like to thank you for your comments. We fully agree that both the incremental capacity analysis (ICA) and the differential voltage analysis (DVA) are effective and classic methods for revealing the degradation mechanisms of batteries.

In our work, we chose these methods not only for their commonality, but for their physical interpretability. Unlike data-driven based approaches, ICA and DVA provide a physical foundation for the health feature extraction of batteries. Therefore, the main innovation and contribution of our work lies in:

(i) Feature Sifting: We employ Kernel Principal Component Analysis (KPCA) to capture these health-related physical features, which can remove data redundancy at the same time.

(ii) Hybrid Deep Learning & Optimization Structure: The core contribution of this work is the integration of the Transformer’s global attention mechanism with LSTM’s local temporal sensitivity, which are further optimized by the NRBO algorithm.

The operation of ICA/DVA is employed to observe battery aging, while the proposed NRBO-optimized Transformer-LSTM framework is applied to learn the behavioral characteristics of battery decay processes with high accuracy and robustness.

**Reviewer#1, Concern # 2:** This paper uses kernel principal component analysis to reduce the dimensionality and remove redundancies of health features. This method is very common.

**Author response:** We acknowledge that Kernel Principal Component Analysis (KPCA) is a well-established technique for dimensionality reduction. However, its application in our study serves as the critical and specific purpose in the proposed hybrid analysis framework for the physical mechanisms and data characteristics of battery health and lifespan.

In practice, the health indicators extracted via ICA/DVA are highly sensitive to noise and often exhibit strong non-linear correlations with each other. Therefore, the operation of KPCA is applied to map the non-linearly correlated features into a higher-dimensional space and retain the most informative indirect health features (IHFs). In addition, the proposed Transformer-LSTM hybrid model is designed to capture complex temporal dependencies. If one feeds raw, redundant features into this network, the risk of overfitting will increase. Accordingly, KPCA acts as a preprocessing stage that simplifies the input space, thereby enhancing the training stability and generalization capability of the subsequent deep learning model.

Overall, KPCA serves as a bridge between physical diagnosis and deep learning, adding physical constraints to the proposed model, which is essential in the practical operation of battery management system (BMS).

**Reviewer#1, Concern # 3:** The author uses the Newton-Raphson optimization algorithm to optimize the hyperparameters of Transformer-LSTM. Where is the innovation?

**Author response:** We appreciate this comment regarding the novelty of using the Newton-Raphson Optimization (NRBO) algorithm.

The innovation of this work does not lie in proposing the Newton-Raphson method itself, but rather in the strategic integration and application of this robust optimization technique to solve the specific challenges of hyperparameter tuning processes of deep learning models for battery RUL prediction. To highlight this purpose, we have revised the Introduction and Methodology sections, framing NRBO not just as a tool, but as a key enabler for maximizing the potential of the hybrid deep learning architecture. In addition, To demonstrate that the choice of NRBO is not arbitrary but superior, we have added comparative experiments in the revised manuscript. The results indicate that NRBO achieves at least **29.51% reduction in RMSE** (as **Table 7** of the revision) and converges at least **1.48 times faster** than the other algorithms (as **Table 10** of the revision), which prove that the specific application of NRBO significantly enhances the model’s robustness and efficiency.

**Reviewer#1, Concern # 4:** This paper validates based on the NASA battery dataset, but this data is too outdated.

**Author response:** We agree that the NASA dataset, released in 2007, represents older battery generation technologies and that relying on it might limit the assessment of the proposed method’s applicability to modern BMS.

To address this concern, we have added experiments using the CALCE Battery Dataset in the revised manuscript, from the following URL:

https://calce.umd.edu/data

As shown in the newly added comparisons, our method maintains high accuracy on CALCE dataset, which proves its generalization capability across different data and battery generations. In addition, we have added a dedicated paragraph in the Discussion section to acknowledge the limitations of laboratory datasets. We clarified that while our method works well on public datasets, future work will focus on validating the model with real-world data to account for more complex noise and variable working conditions.

**Reviewer#1, Concern # 5:** The author uses a 70% training set ratio in the experiment, which is too much.

**Author response:** We thank the reviewer for this comment, and we acknowledge that a 70% training ratio might be relatively high, which might limit the size of the test set. Our initial choice of 70% was driven by the data-hungry nature of the proposed deep learning model. With hundreds number of battery cycles available in NASA dataset, we aimed to provide sufficient samples for the model to learn the complex non-linear degradation features without underfitting.

**Reviewer#1, Concern # 6:** The author needs to compare with the methods in recent high-level research works.

**Author response:** We sincerely thank the reviewer for this suggestion. In the revised manuscript, we have added comparisons with some representative studies published in recent three years. We used the same experimental settings to ensure a fair comparison. The results are summarized in Table 7 and Fig. 6 to Fig 10 of the revised manuscript.

**Reviewer#2, Concern # 1:** **According to the data in Table 2, the Pearson coefficients of HF1 (peak height of the IC curve) for batteries B0005 and B0007 reached 0.9098 and 0.9731 respectively. In many literature, a coefficient above 0.9 has been regarded as extremely strong correlation. The author only excluded HF1 in the text based on the reason of "comparatively lower", which is not sufficient. Please provide a detailed explanation as to whether the exclusion of HF1 is because of its volatility across different batteries (such as B0031 with only 0.6325) affecting the generalization ability of the model.**

**Author response:** We sincerely thank you for your comments. We acknowledge that our original statement “comparatively lower” is not sufficient. To respond to your concern, we provide a detailed clarification for the exclusion of HF1:

As Table 3, HF1 exhibits strong correlations with SOH for batteries B0005 (0.9098) and B0007 (0.9731). However, for other cells-particularly B0031-its Pearson correlation coefficient drops significantly to **0.6325**, well below the commonly accepted threshold of 0.9 for a strong linear relationship. This substantial performance fluctuation across different batteries (ranging from 0.9731 to 0.6325) is the core reason for our decision to exclude HF1.

Our research objective is to develop a **robust and generalizable** SOH estimation framework. The high correlation of HF1 appears to be highly dependent on specific operating conditions. For instance, B0005 and B0007 were aged at 24°C under a 2A discharge rate-whereas under different aging conditions (e.g., B0031 aged at 43°C with a 4A discharge rate), its linear relationship with capacity degrades obviously, which suggests that HF1 may be overly sensitive to external factors such as temperature and C-rate, and its physical interpretation may become unstable under complex, multi-factor coupled degradation processes.

In contrast, the retained features HF2 to HF4 demonstrate **consistent and stable** high correlations across all four battery types (B0005, B0007, B0029, and B0031). These features capture a more comprehensive picture on the degradation mechanisms of batteries, thereby offering greater reliability and cross-dataset adaptability as model inputs.

In summary, the exclusion of HF1 is not a dismissal of its value in specific scenarios, but a deliberate choice to **prioritize overall model robustness and generalization capability**. We have revised Section 2.3 in the manuscript to clarify this point, explicitly highlighting HF1’s low correlation on B0031, its potential negative impact on generalization, and our guiding principle that “stability across diverse datasets outweighs peak performance on a single dataset.

**Reviewer#2, Concern # 2:** **In the experimental evaluation, the author only used the NASA lithium-ion battery cycling dataset and did not conduct cross-validation across datasets or individual batteries, making it difficult to fully prove the generalization ability of the model. It is recommended to add other datasets or conduct hold-out experiments with the same dataset.**

**Author response:** We agree that the NASA dataset, released in 2007, represents older battery generation technologies and that relying on it might limit the assessment of the proposed method’s applicability to modern BMS.

To address this concern, we have added experiments using the CALCE Battery Dataset in the revised manuscript, from the following URL:

https://calce.umd.edu/data

Specifically, we applied our method to two cells from the CALCE dataset, that is, CS2_35 and CS2_36, and used the same train/test split strategy as employed for the NASA dataset. The results demonstrate that our NRBO-Transformer-LSTM model also achieves good performance on the CALCE dataset, which confirms that the proposed framework and hybrid prediction model are not only effective on the NASA dataset but also successfully generalize to other publicly available datasets with different cell chemistries and aging conditions, thereby significantly enhancing the robustness and universality of our approach.

We have thoroughly updated Section 4 in the revised manuscript to include these new comparisons and analysis, along with updated figures, tables, and corresponding discussions.

**Reviewer#2, Concern # 3: For the experiments of batteries B0029 and B0031, the sample sizes of the training and test sets for these two batteries are insufficient. Moreover, the Transformer encoder and LSTM network both contain complex gating mechanisms and multi-head attention mechanisms, with a large number of parameters. Training such a complex hybrid deep learning model on a training set with only 27 time steps/samples has a very high risk of overfitting. The author needs to provide a detailed explanation on how to ensure the generalization ability of the model under such a small sample size, or explore why the overall life cycle data of these two batteries is so short.**

**Author response:** We appreciate this comment regarding the insufficient sample size of data and the overfitting risk of the model. In response to your concern, we provide the following clarifications:

The lifecycle data of batteries B0029 and B0031 are from NASA public dataset. As shown in Table 1 of our paper, these two cells underwent accelerated aging tests under the conditions of 43°C and a 4A discharge current. These operating conditions accelerated the battery aging. Consequently, the number of available data points is limited.

We agree that training such a complex model on a dataset with only 27 samples brings the overfitting risk. To mitigate this risk and ensure the model’s generalization capability, we have implemented several measures in our model design and training process:

(i) We did not use the raw charging data directly. Instead, we first extracted health-related features (HFs) correlated with aging mechanisms using ICA/DVA methods. Subsequently, we employed KPCA to compress these features into a single, most informative Indirect Health Feature (IHF). This process can reduce the dimensionality and redundancy of the input data, thereby alleviating the small-sample problem.
(ii) Our hybrid model incorporates L2 regularization. Moreover, the regularization coefficient of the model is automatically optimized by the NRBO algorithm. As a result, the model can suppress complex weights during training and achieve an optimal balance between fitting capability and generalization ability.

(iii) The adopted NRBO algorithm contains a trap avoidance operator (TAO), which can help the model escape local optima and find solutions with better generalization potential.

In summary, although the data volume for B0029 and B0031 is limited, our model effectively controls the risk of overfitting by feature dimensionality reduction, automated optimization of regularization strength, and an advanced hyperparameter search strategy. The experimental results confirm that: even under small-sample conditions, the proposed model achieves stable prediction performance that is significantly superior to other benchmark methods.

**Reviewer#2, Concern # 4:** **In the introduction section, the author correctly pointed out that some existing deep learning models are limited in real-time deployment in resource-constrained battery management systems (BMS) due to high computational costs. However, the method proposed in this paper sequentially combines KPCA dimensionality reduction, Transformer encoder, LSTM time modeling, and NRBO hyperparameter optimization algorithms. The text completely lacks a quantitative assessment of the computational complexity or inference time of the proposed model.**

**Author response:** We sincerely thank you for this insightful comment. To respond to your concern regarding the lack of assessment of computational complexity or inference time, we have added a subsection (Section 4.5) on computational efficiency analysis, along with the computational time comparison of different optimization algorithms. Specifically:

We compare the computational time of the proposed NRBO algorithm against GWO and WOA under identical experimental settings, as shown in Table 10. The results demonstrate that NRBO achieves the lowest computational time due to the Newton-Raphson-based search rule (NRSR) and the trap avoidance operator (TAO). These mechanisms not only enable NRBO to avoid the premature convergence that plagues GWO but also accelerate convergence, leading to reduced runtime.

**Reviewer#2, Concern # 5:** **In Table 3 (Table 3), the first column lacks a header name. Based on the context data, this list header should be named "Battery". Please complete it. Additionally, the format and grammar of the article still need to be further optimized.**

**Author response:** Thank you very much for your suggest. In the revised manuscript, we have added the missing column header “Battery Number” to the first column of Table 5 to ensure the table is clear and complete. In addition, we have thoroughly proofread and refined the entire manuscript. We sincerely appreciate your help in improving the quality of our work.
