# Supplementary material for: Data-driven and feature-sifting based state-of-health estimation for li-ion batteries using KPCA and NRBO-optimized transformer-LSTM hybrid model
Source: PLoS One. 2026 Oct 5;21(10):e0359015. doi: 10.1371/journal.pone.0359015 (PMC13638052; doi:10.1371/journal.pone.0359015)
Supplement: Peer review history

# Peer review: Decision Letter 0

**DOI:** 10.1371/journal.pone.0359015.r001

**Authors:** Shaheer Ansari

15 Apr 2026

PONE-D-26-13461

Data-Driven and Feature-Sifting Based State-of-Health Estimation for Li-Ion Batteries Using KPCA and NRBO-Optimized Transformer-LSTM Hybrid Model

PLOS One

Dear Dr. Chen,

Thank you for submitting your manuscript to PLOS ONE. After careful consideration, we feel that it has merit but does not fully meet PLOS ONE’s publication criteria as it currently stands. Therefore, we invite you to submit a revised version of the manuscript that addresses the points raised during the review process.

Please submit your revised manuscript by May 29 2026 11:59PM. If you will need more time than this to complete your revisions, please reply to this message or contact the journal office at plosone@plos.org. When you're ready to submit your revision, log on to https://www.editorialmanager.com/pone/ and select the 'Submissions Needing Revision' folder to locate your manuscript file.

Please include the following items when submitting your revised manuscript:

- A letter that responds to each point raised by the academic editor and reviewer(s). You should upload this letter as a separate file labeled 'Response to Reviewers'.
- A marked-up copy of your manuscript that highlights changes made to the original version. You should upload this as a separate file labeled 'Revised Manuscript with Track Changes'.
- An unmarked version of your revised paper without tracked changes. You should upload this as a separate file labeled 'Manuscript'.

If you would like to make changes to your financial disclosure, please include your updated statement in your cover letter. Guidelines for resubmitting your figure files are available below the reviewer comments at the end of this letter.

If applicable, we recommend that you deposit your laboratory protocols in protocols.io to enhance the reproducibility of your results. Protocols.io assigns your protocol its own identifier (DOI) so that it can be cited independently in the future. For instructions see: https://journals.plos.org/plosone/s/submission-guidelines#loc-laboratory-protocols. Additionally, PLOS ONE offers an option for publishing peer-reviewed Lab Protocol articles, which describe protocols hosted on protocols.io. Read more information on sharing protocols at https://plos.org/protocols?utm_medium=editorial-email&utm_source=authorletters&utm_campaign=protocols.

As the corresponding author, your ORCID iD is verified in the submission system and will appear in the published article. PLOS supports the use of ORCID, and we encourage all coauthors to register for an ORCID iD and use it as well. Please encourage your coauthors to verify their ORCID iD within the submission system before final acceptance, as unverified ORCID iDs will not appear in the published article. *Only*  the individual author can complete the verification step; PLOS staff *cannot*  verify ORCID iDs on behalf of authors.

We look forward to receiving your revised manuscript.

Kind regards,

Shaheer Ansari

Academic Editor

PLOS One

**Journal Requirements:**

When submitting your revision, we need you to address these additional requirements.

1. Please ensure that your manuscript meets PLOS ONE's style requirements, including those for file naming. The PLOS ONE style templates can be found at

https://journals.plos.org/plosone/s/file?id=wjVg/PLOSOne_formatting_sample_main_body.pdf and

https://journals.plos.org/plosone/s/file?id=ba62/PLOSOne_formatting_sample_title_authors_affiliations.pdf

2. Please note that PLOS One has specific guidelines on code sharing for submissions in which author-generated code underpins the findings in the manuscript. In these cases, we expect all author-generated code to be made available without restrictions upon publication of the work. Please review our guidelines at https://journals.plos.org/plosone/s/materials-and-software-sharing#loc-sharing-code and ensure that your code is shared in a way that follows best practice and facilitates reproducibility and reuse.

3. Thank you for stating the following in the Financial Disclosure section:

“State Grid Sichuan Electric Power Company Technology Project, Grant No. 521909240001'

We note that you received funding from a commercial source: State Grid Sichuan Electric Power Company Technology

Please provide an amended Competing Interests Statement that explicitly states this commercial funder, along with any other relevant declarations relating to employment, consultancy, patents, products in development, marketed products, etc.

Within this Competing Interests Statement, please confirm that this does not alter your adherence to all PLOS ONE policies on sharing data and materials by including the following statement: 'This does not alter our adherence to PLOS ONE policies on sharing data and materials.” (as detailed online in our guide for authors http://journals.plos.org/plosone/s/competing-interests).  If there are restrictions on sharing of data and/or materials, please state these. Please note that we cannot proceed with consideration of your article until this information has been declared.

Please include your amended Competing Interests Statement within your cover letter. We will change the online submission form on your behalf.

4. We note that one or more of the authors are employed by a commercial company: State Grid Neijiang Power Supply Co., Ltd

1. Please provide an amended Funding Statement declaring this commercial affiliation, as well as a statement regarding the Role of Funders in your study. If the funding organization did not play a role in the study design, data collection and analysis, decision to publish, or preparation of the manuscript and only provided financial support in the form of authors' salaries and/or research materials, please review your statements relating to the author contributions, and ensure you have specifically and accurately indicated the role(s) that these authors had in your study. You can update author roles in the Author Contributions section of the online submission form.

Please also include the following statement within your amended Funding Statement.

“The funder provided support in the form of salaries for authors [insert relevant initials], but did not have any additional role in the study design, data collection and analysis, decision to publish, or preparation of the manuscript. The specific roles of these authors are articulated in the ‘author contributions’ section.”

If your commercial affiliation did play a role in your study, please state and explain this role within your updated Funding Statement.

2. Please also provide an updated Competing Interests Statement declaring this commercial affiliation along with any other relevant declarations relating to employment, consultancy, patents, products in development, or marketed products, etc.

Within your Competing Interests Statement, please confirm that this commercial affiliation does not alter your adherence to all PLOS ONE policies on sharing data and materials by including the following statement: 'This does not alter our adherence to  PLOS ONE policies on sharing data and materials.” (as detailed online in our guide for authors http://journals.plos.org/plosone/s/competing-interests) . If this adherence statement is not accurate and  there are restrictions on sharing of data and/or materials, please state these. Please note that we cannot proceed with consideration of your article until this information has been declared.

Please include both an updated Funding Statement and Competing Interests Statement in your cover letter. We will change the online submission form on your behalf.

5. If the reviewer comments include a recommendation to cite specific previously published works, please review and evaluate these publications to determine whether they are relevant and should be cited. There is no requirement to cite these works unless the editor has indicated otherwise.

[Note: HTML markup is below. Please do not edit.]

Reviewers' comments:

Reviewer's Responses to Questions

**Comments to the Author**

1. Is the manuscript technically sound, and do the data support the conclusions?

The manuscript must describe a technically sound piece of scientific research with data that supports the conclusions. Experiments must have been conducted rigorously, with appropriate controls, replication, and sample sizes. The conclusions must be drawn appropriately based on the data presented.

Reviewer #1: Yes

Reviewer #2: Partly

**********

2. Has the statistical analysis been performed appropriately and rigorously?

Reviewer #1: Yes

Reviewer #2: Yes

**********

3. Have the authors made all data underlying the findings in their manuscript fully available?

The PLOS Data policy requires authors to make all data underlying the findings described in their manuscript fully available without restriction, with rare exception (please refer to the Data Availability Statement in the manuscript PDF file). The data should be provided as part of the manuscript or its supporting information, or deposited to a public repository. For example, in addition to summary statistics, the data points behind means, medians and variance measures should be available. If there are restrictions on publicly sharing data—e.g. participant privacy or use of data from a third party—those must be specified.

Reviewer #1: Yes

Reviewer #2: Yes

**********

4. Is the manuscript presented in an intelligible fashion and written in standard English?

PLOS ONE does not copyedit accepted manuscripts, so the language in submitted articles must be clear, correct, and unambiguous. Any typographical or grammatical errors should be corrected at revision, so please note any specific errors here.

Reviewer #1: Yes

Reviewer #2: Yes

**********

5. Review Comments to the Author

Please use the space provided to explain your answers to the questions above. You may also include additional comments for the author, including concerns about dual publication, research ethics, or publication ethics. (Please upload your review as an attachment if it exceeds 20,000 characters)

Reviewer #1: This study establishes a feature-sifting analysis framework combining ICA, DVA, and KPCA for health feature extraction, and employs the NRBO algorithm to optimize hyperparameters of a Transformer-LSTM model.

1. This research work employs incremental capacity analysis and differential voltage analysis to extract health features, but these methods are quite common.

2. This paper uses kernel principal component analysis to reduce the dimensionality and remove redundancies of health features. This method is very common.

3. The author uses the Newton-Raphson optimization algorithm to optimize the hyperparameters of Transformer-LSTM. Where is the innovation?

4. This paper validates based on the NASA battery dataset, but this data is too outdated.

5. The author uses a 70% training set ratio in the experiment, which is too much.

6. The author needs to compare with the methods in recent high-level research works.

Reviewer #2: 1. According to the data in Table 2, the Pearson coefficients of HF1 (peak height of the IC curve) for batteries B0005 and B0007 reached 0.9098 and 0.9731 respectively. In many literature, a coefficient above 0.9 has been regarded as extremely strong correlation. The author only excluded HF1 in the text based on the reason of "comparatively lower", which is not sufficient. Please provide a detailed explanation as to whether the exclusion of HF1 is because of its volatility across different batteries (such as B0031 with only 0.6325) affecting the generalization ability of the model. 2. In the experimental evaluation, the author only used the NASA lithium-ion battery cycling dataset and did not conduct cross-validation across datasets or individual batteries, making it difficult to fully prove the generalization ability of the model. It is recommended to add other datasets or conduct hold-out experiments with the same dataset. 3. For the experiments of batteries B0029 and B0031, the sample sizes of the training and test sets for these two batteries are insufficient. Moreover, the Transformer encoder and LSTM network both contain complex gating mechanisms and multi-head attention mechanisms, with a large number of parameters. Training such a complex hybrid deep learning model on a training set with only 27 time steps/samples has a very high risk of overfitting. The author needs to provide a detailed explanation on how to ensure the generalization ability of the model under such a small sample size, or explore why the overall life cycle data of these two batteries is so short. 4. In the introduction section, the author correctly pointed out that some existing deep learning models are limited in real-time deployment in resource-constrained battery management systems (BMS) due to high computational costs. However, the method proposed in this paper sequentially combines KPCA dimensionality reduction, Transformer encoder, LSTM time modeling, and NRBO hyperparameter optimization algorithms. The text completely lacks a quantitative assessment of the computational complexity or inference time of the proposed model. 5. In Table 3 (Table 3), the first column lacks a header name. Based on the context data, this list header should be named "Battery". Please complete it. Additionally, the format and grammar of the article still need to be further optimized.

**********

6. PLOS authors have the option to publish the peer review history of their article (what does this mean?). If published, this will include your full peer review and any attached files.

If you choose “no”, your identity will remain anonymous but your review may still be made public.

**Do you want your identity to be public for this peer review?**  For information about this choice, including consent withdrawal, please see our Privacy Policy.

Reviewer #1: No

Reviewer #2: No

**********

[NOTE: If reviewer comments were submitted as an attachment file, they will be attached to this email and accessible via the submission site. Please log into your account, locate the manuscript record, and check for the action link "View Attachments". If this link does not appear, there are no attachment files.]

To ensure your figures meet our technical requirements, please review our figure guidelines: https://journals.plos.org/plosone/s/figures

You may also use PLOS’s free figure tool, NAAS, to help you prepare publication quality figures: https://journals.plos.org/plosone/s/figures#loc-tools-for-figure-preparation.

NAAS will assess whether your figures meet our technical requirements by comparing each figure against our figure specifications.

Attachment Submitted filename: review comment.docx

# Author comment: Author response to Decision Letter 1

**DOI:** 10.1371/journal.pone.0359015.r002

22 Jun 2026

Reviewer#1, Concern # 1: This research work employs incremental capacity analysis and differential voltage analysis to extract health features, but these methods are quite common.

Author response: We would like to thank you for your comments. We fully agree that both the incremental capacity analysis (ICA) and the differential voltage analysis (DVA) are effective and classic methods for revealing the degradation mechanisms of batteries.

In our work, we chose these methods not only for their commonality, but for their physical interpretability. Unlike data-driven based approaches, ICA and DVA provide a physical foundation for the health feature extraction of batteries. Therefore, the main innovation and contribution of our work lies in:

(i) Feature Sifting: We employ Kernel Principal Component Analysis (KPCA) to capture these health-related physical features, which can remove data redundancy at the same time.

(ii) Hybrid Deep Learning & Optimization Structure: The core contribution of this work is the integration of the Transformer’s global attention mechanism with LSTM’s local temporal sensitivity, which are further optimized by the NRBO algorithm.

The operation of ICA/DVA is employed to observe battery aging, while the proposed NRBO-optimized Transformer-LSTM framework is applied to learn the behavioral characteristics of battery decay processes with high accuracy and robustness.

Reviewer#1, Concern # 2: This paper uses kernel principal component analysis to reduce the dimensionality and remove redundancies of health features. This method is very common.

Author response: We acknowledge that Kernel Principal Component Analysis (KPCA) is a well-established technique for dimensionality reduction. However, its application in our study serves as the critical and specific purpose in the proposed hybrid analysis framework for the physical mechanisms and data characteristics of battery health and lifespan.

In practice, the health indicators extracted via ICA/DVA are highly sensitive to noise and often exhibit strong non-linear correlations with each other. Therefore, the operation of KPCA is applied to map the non-linearly correlated features into a higher-dimensional space and retain the most informative indirect health features (IHFs). In addition, the proposed Transformer-LSTM hybrid model is designed to capture complex temporal dependencies. If one feeds raw, redundant features into this network, the risk of overfitting will increase. Accordingly, KPCA acts as a preprocessing stage that simplifies the input space, thereby enhancing the training stability and generalization capability of the subsequent deep learning model.

Overall, KPCA serves as a bridge between physical diagnosis and deep learning, adding physical constraints to the proposed model, which is essential in the practical operation of battery management system (BMS).

Reviewer#1, Concern # 3: The author uses the Newton-Raphson optimization algorithm to optimize the hyperparameters of Transformer-LSTM. Where is the innovation?

Author response: We appreciate this comment regarding the novelty of using the Newton-Raphson Optimization (NRBO) algorithm.

The innovation of this work does not lie in proposing the Newton-Raphson method itself, but rather in the strategic integration and application of this robust optimization technique to solve the specific challenges of hyperparameter tuning processes of deep learning models for battery RUL prediction. To highlight this purpose, we have revised the Introduction and Methodology sections, framing NRBO not just as a tool, but as a key enabler for maximizing the potential of the hybrid deep learning architecture. In addition, To demonstrate that the choice of NRBO is not arbitrary but superior, we have added comparative experiments in the revised manuscript. The results indicate that NRBO achieves at least 29.51% reduction in RMSE (as Table 7 of the revision) and converges at least 1.48 times faster than the other algorithms (as Table 10 of the revision), which prove that the specific application of NRBO significantly enhances the model’s robustness and efficiency.

Reviewer#1, Concern # 4: This paper validates based on the NASA battery dataset, but this data is too outdated.

Author response: We agree that the NASA dataset, released in 2007, represents older battery generation technologies and that relying on it might limit the assessment of the proposed method’s applicability to modern BMS.

To address this concern, we have added experiments using the CALCE Battery Dataset in the revised manuscript, from the following URL:

https://calce.umd.edu/data

As shown in the newly added comparisons, our method maintains high accuracy on CALCE dataset, which proves its generalization capability across different data and battery generations. In addition, we have added a dedicated paragraph in the Discussion section to acknowledge the limitations of laboratory datasets. We clarified that while our method works well on public datasets, future work will focus on validating the model with real-world data to account for more complex noise and variable working conditions.

Reviewer#1, Concern # 5: The author uses a 70% training set ratio in the experiment, which is too much.

Author response: We thank the reviewer for this comment, and we acknowledge that a 70% training ratio might be relatively high, which might limit the size of the test set. Our initial choice of 70% was driven by the data-hungry nature of the proposed deep learning model. With hundreds number of battery cycles available in NASA dataset, we aimed to provide sufficient samples for the model to learn the complex non-linear degradation features without underfitting.

Reviewer#1, Concern # 6: The author needs to compare with the methods in recent high-level research works.

Author response: We sincerely thank the reviewer for this suggestion. In the revised manuscript, we have added comparisons with some representative studies published in recent three years. We used the same experimental settings to ensure a fair comparison. The results are summarized in Table 7 and Fig. 6 to Fig 10 of the revised manuscript.

Reviewer#2, Concern # 1: According to the data in Table 2, the Pearson coefficients of HF1 (peak height of the IC curve) for batteries B0005 and B0007 reached 0.9098 and 0.9731 respectively. In many literature, a coefficient above 0.9 has been regarded as extremely strong correlation. The author only excluded HF1 in the text based on the reason of "comparatively lower", which is not sufficient. Please provide a detailed explanation as to whether the exclusion of HF1 is because of its volatility across different batteries (such as B0031 with only 0.6325) affecting the generalization ability of the model.

Author response: We sincerely thank you for your comments. We acknowledge that our original statement “comparatively lower” is not sufficient. To respond to your concern, we provide a detailed clarification for the exclusion of HF1:

As Table 3, HF1 exhibits strong correlations with SOH for batteries B0005 (0.9098) and B0007 (0.9731). However, for other cells-particularly B0031-its Pearson correlation coefficient drops significantly to 0.6325, well below the commonly accepted threshold of 0.9 for a strong linear relationship. This substantial performance fluctuation across different batteries (ranging from 0.9731 to 0.6325) is the core reason for our decision to exclude HF1.

Our research objective is to develop a robust and generalizable SOH estimation framework. The high correlation of HF1 appears to be highly dependent on specific operating conditions. For instance, B0005 and B0007 were aged at 24°C under a 2A discharge rate-whereas under different aging conditions (e.g., B0031 aged at 43°C with a 4A discharge rate), its linear relationship with capacity degrades obviously, which suggests that HF1 may be overly sensitive to external factors such as temperature and C-rate, and its physical interpretation may become unstable under complex, multi-factor coupled degradation processes.

In contrast, the retained features HF2 to HF4 demonstrate consistent and stable high correlations across all four battery types (B0005, B0007, B0029, and B0031). These features capture a more comprehensive picture on the degradation mechanisms of batteries, thereby offering greater reliability and cross-dataset adaptability as model inputs.

In summary, the exclusion of HF1 is not a dismissal of its value in specific scenarios, but a deliberate choice to prioritize overall model robustness and generalization capability. We have revised Section 2.3 in the manuscript to clarify this point, explicitly highlighting HF1’s low correlation on B0031, its potential negative impact on generalization, and our guiding principle that “stability across diverse datasets outweighs peak performance on a single dataset.

Reviewer#2, Concern # 2: In the experimental evaluation, the author only used the NASA lithium-ion battery cycling dataset and did not conduct cross-validation across datasets or individual batteries, making it difficult to fully prove the generalization ability of the model. It is recommended to add other datasets or conduct hold-out experiments with the same dataset.

Author response: We agree that the NASA dataset, released in 2007, represents older battery generation technologies and that relying on it might limit the assessment of the proposed method’s applicability to modern BMS.

To address this concern, we have added experiments using the CALCE Battery Dataset in the revised manuscript, from the following URL:

https://calce.umd.edu/data

Specifically, we applied our method to two cells from the CALCE dataset, that is, CS2_35 and CS2_36, and used the same train/test split strategy as employed for the NASA dataset. The results demonstrate that our NRBO-Transformer-LSTM model also achieves good performance on the CALCE dataset, which confirms that the proposed framework and hybrid prediction model are not only effective on the NASA dataset but also successfully generalize to other publicly available datasets with different cell chemistries and aging conditions, thereby significantly enhancing the robustness and universality of our approach.

We have thoroughly updated Section 4 in the revised manuscript to include these new comparisons and analysis, along with updated figures, tables, and corresponding discussions.

Reviewer#2, Concern # 3: For the experiments of batteries B0029 and B0031, the sample sizes of the training and test sets for these two batteries are insufficient. Moreover, the Transformer encoder and LSTM network both contain complex gating mechanisms and multi-head attention mechanisms, with a large number of parameters. Training such a complex hybrid deep learning model on a training set with only 27 time steps/samples has a very high risk of overfitting. The author needs to provide a detailed explanation on how to ensure the generalization ability of the model under such a small sample size, or explore why the overall life cycle data of these two batteries is so short.

Author response: We appreciate this comment regarding the insufficient sample size of data and the overfitting risk of the model. In response to your concern, we provide the following clarifications:

The lifecycle data of batteries B0029 and B0031 are from NASA public dataset. As shown in Table 1 of our paper, these two cells underwent accelerated aging tests under the conditions of 43°C and a 4A discharge current. These operating conditions accelerated the battery aging. Consequently, the number of available data points is limited.

We agree that training such a complex model on a dataset with only 27 samples brings the overfitting risk. To mitigate this risk and ensure the model’s generalization capability, we have implemented several measures in our model design and training process:

(i)We did not use the raw charging data directly. Instead, we first extracted health-related features (HFs) correlated with aging mechanisms using ICA/DVA methods. Subsequently, we employed KPCA to compress these features into a single, most informative Indirect Health Feature (IHF). This process can reduce the dimensionality and redundancy of the input data, thereby alleviating the small-sample problem.

(ii)Our hybrid model incorporates L2 regularization. Moreover, the regularization coefficient of the model is automatically optimized by the NRBO algorithm. As a result, the model can suppress complex weights during training and achieve an optimal balance between fitting capability and generalization ability.

(iii) The adopted NRBO algorithm contains a trap avoidance operator (TAO), which can help the model escape local optima and find solutions with better generalization potential.

In summary, although the data volume for B0029 and B0031 is limited, our model effectively controls the risk of overfitting by feature dimensionality reduction, automated optimization of regularization strength, and an advanced hyperparameter search strategy. The experimental results confirm that: even under small-sample conditions, the proposed model achieves stable prediction performance that is significantly superior to other benchmark methods.

Reviewer#2, Concern # 4: In the introduction section, the author correctly pointed out that some existing deep learning models are limited in real-time deployment in resource-constrained battery management systems (BMS) due to high computational costs. However, the method proposed in this paper sequentially combines KPCA dimensionality reduction, Transformer encoder, LSTM time modeling, and NRBO hyperparameter optimization algorithms. The text completely lacks a quantitative assessment of the computational complexity or inference time of the proposed model.

Author response: We sincerely thank you for this insightful comment. To respond to your concern regarding the lack of assessment of computational complexity or inference time, we have added a subsection (Section 4.5) on computational efficiency analysis, along with the computational time comparison of different optimization algorithms. Specifically:

We compare the computational time of the proposed NRBO algorithm against GWO and WOA under identical experimental settings, as shown in Table 10. The results demonstrate that NRBO achieves the lowest computational time due to the Newton-Raphson-based search rule (NRSR) and the trap avoidance operator (TAO). These mechanisms not only enable NRBO to avoid the premature convergence that plagues GWO but also accelerate convergence, leading to reduced runtime.

Reviewer#2, Concern # 5: In Table 3 (Table 3), the first column lacks a header name. Based on the context data, this list header should be named "Battery". Please complete it. Additionally, the format and grammar of the article still need to be further optimized.

Author response: Thank you very much for your suggest. In the revised manuscript, we have added the missing column header “Battery Number” to the first column of Table 5 to ensure the table is clear and complete. In addition, we have thoroughly proofread and refined the entire manuscript. We sincerely appreciate your help in improving the quality of our work.

Attachment Submitted filename: Response Letter20260524.docx

# Peer review: Decision Letter 1

**DOI:** 10.1371/journal.pone.0359015.r003

**Authors:** Shaheer Ansari

8 Sep 2026

Data-Driven and Feature-Sifting Based State-of-Health Estimation for Li-Ion Batteries Using KPCA and NRBO-Optimized Transformer-LSTM Hybrid Model

PONE-D-26-13461R1

Dear Dr. Chen,

We’re pleased to inform you that your manuscript has been judged scientifically suitable for publication and will be formally accepted for publication once it meets all outstanding technical requirements.

Within one week, you’ll receive an e-mail detailing the required amendments. When these have been addressed, you’ll receive a formal acceptance letter and your manuscript will be scheduled for publication.

An invoice will be generated when your article is formally accepted. Please note, if your institution has a publishing partnership with PLOS and your article meets the relevant criteria, all or part of your publication costs will be covered. Please make sure your user information is up-to-date by logging into Editorial Manager at Editorial Manager® and clicking the ‘Update My Information' link at the top of the page. For questions related to billing, please contact billing support.

If your institution or institutions have a press office, please notify them about your upcoming paper to help maximize its impact. If they’ll be preparing press materials, please inform our press team as soon as possible -- no later than 48 hours after receiving the formal acceptance. Your manuscript will remain under strict press embargo until 2 pm Eastern Time on the date of publication. For more information, please contact onepress@plos.org.

Kind regards,

Shaheer Ansari

Academic Editor

PLOS One

**********

# Editor report: Acceptance letter

**DOI:** 10.1371/journal.pone.0359015.r004

**Authors:** Shaheer Ansari

PONE-D-26-13461R1

PLOS One

Dear Dr. Chen,

I'm pleased to inform you that your manuscript has been deemed suitable for publication in PLOS One. Congratulations! Your manuscript is now being handed over to our production team.

At this stage, our production department will prepare your paper for publication. This includes ensuring the following:

* All references, tables, and figures are properly cited

* All relevant supporting information is included in the manuscript submission,

* There are no issues that prevent the paper from being properly typeset

You will receive further instructions from the production team, including instructions on how to review your proof when it is ready. Please keep in mind that we are working through a large volume of accepted articles, so please give us a few days to review your paper and let you know the next and final steps.

Lastly, if your institution or institutions have a press office, please let them know about your upcoming paper now to help maximize its impact. If they'll be preparing press materials, please inform our press team within the next 48 hours. Your manuscript will remain under strict press embargo until 2 pm Eastern Time on the date of publication. For more information, please contact onepress@plos.org.

You will receive an invoice from PLOS for your publication fee after your manuscript has reached the completed accept phase. If you receive an email requesting payment before acceptance or for any other service, this may be a phishing scheme. Learn how to identify phishing emails and protect your accounts at https://explore.plos.org/phishing.

If we can help with anything else, please email us at customercare@plos.org.

Thank you for submitting your work to PLOS One and supporting open access.

Kind regards,

PLOS One Editorial Office Staff

on behalf of

Dr. Shaheer Ansari

Academic Editor

PLOS One
